# Recent advances and challenges in hydrogel-based delivery of immunomodulatory strategies for diabetic wound healing

**DOI:** 10.7150/thno.117949

**Published:** 2026-01-01

**Authors:** Longyu Du, Chuanlu Lin, Haifeng Hu, Yanzhi Zhao, Jiewen Liao, Fawwaz Al-Smadi, Bobin Mi, Yiqiang Hu, Guohui Liu

**Affiliations:** 1 Department of Orthopedics, Union Hospital, Tongji Medical College, Huazhong University of Science and Technology, Wuhan 430022, China.; 2 Hubei Key Laboratory of Regenerative Medicine and Multi-disciplinary Translational Research (Huazhong University of Science and Technology), Wuhan 430022, China.

**Keywords:** diabetic wounds, immunomodulation, hydrogels, extracellular vesicles, biomaterials

## Abstract

Chronic wounds associated with diabetes present considerable clinical hurdles, primarily due to delayed tissue repair and dysregulated immune activity. The imbalance in immune responses, including impaired macrophage polarization, excessive neutrophil activation, and oxidative stress, further hampers the healing process. The application of immunomodulatory biologics as a novel treatment method for diabetic wounds often yields limited results due to rapid degradation and lack of targeting. Hydrogels not only prevent rapid drug degradation but also allow for conditional responsiveness and targeted delivery. Therefore, hydrogels loaded with immunomodulatory biologics emerge as a promising strategy, offering the capacity to reshape the immune milieu and promote regenerative outcomes. This review first outlines the role of immune system during the healing processes in normal and diabetic wounds. It then discusses the latest advancements in hydrogel delivery systems as part of immune-modulatory interventions, wherein hydrogels serve as pivotal carriers for (i) cell delivery, such as stem cells and macrophages; (ii) extracellular vesicles derived from both cellular and tissue sources, as well as extracellular vesicle mimetics; and (iii) bioactive substances, including oxygen-releasing microspheres, nanomaterials, and cytokines. Finally, this review focuses on the limitations of modulating immune responses in diabetic wound healing and proposes potential future directions.

## 1. Introduction

Diabetes mellitus is a widespread condition hallmarked by chronic hyperglycemia and its associated range of complications, including vasculopathy, neuropathy, nephropathy, retinopathy, and chronic wounds. According to the International Diabetes Federation's 2021 report, over 500 million people worldwide are living with diabetes, and this number is projected to exceed 780 million by 2045 1. Among the most serious complications are diabetic wounds, particularly those affecting the lower extremities, which can lead to amputation and even death. It is estimated that nearly one-third of individuals with diabetes will develop a diabetic foot ulcer during their lifetime, and approximately 20% of these patients will require amputation 2. Diabetic wounds impose a substantial physical, emotional, and financial burden, highlighting the urgent need for effective and affordable treatment strategies 1. Importantly, patients with type 1 diabetes (T1D) and type 2 diabetes (T2D), as well as those with neuropathic or ischemic complications, may exhibit distinct wound pathologies and immune profiles, which can critically influence therapeutic outcomes.

A wide range of therapeutic strategies has been developed for diabetic wound management, including debridement, infection control, glycemic regulation, hyperbaric oxygen therapy, negative pressure wound therapy, energy-based interventions (e.g., pulsed electromagnetic fields, shockwaves, and lasers), and topical drug delivery. Notably, wound dressings have been a cornerstone of treatment for more than two millennia 3. Advances in biology and materials science have led to the development of innovative dressings composed of natural polymers, synthetic polymers, or hybrid materials. Following Winter's pivotal discovery in 1962 that moist environments accelerate wound healing, there has been rapid progress in the design of moisture-retaining dressings, such as hydrocolloids and hydrogels.

An ideal wound dressing should provide adequate mechanical strength, excellent biocompatibility, and non-toxicity. It should be easy to replace, non-adherent to the wound bed, absorbent without leaking, and capable of maintaining a moist healing environment. Additionally, it should offer antimicrobial protection, reduce pain, enable real-time monitoring, support tissue regeneration, and deliver therapeutic agents in a controlled manner 4-8. Compared to conventional dressings, hydrogels offer distinct advantages by maintaining a moist environment, reducing local temperature, and allowing customization of their physical and chemical properties to suit various wound types. Their composition and crosslinking mechanisms can be tailored to modulate biological responses and enhance healing. Furthermore, hydrogels support targeted and responsive delivery of cells, biologics, and other therapeutics 9-12. Their physicochemical characteristics—such as composition, dimensionality, surface morphology, and porosity—can influence local cell behavior, while hydrogel-based delivery of bioactive agents has been shown to modulate immune responses, stimulate angiogenesis, and control infection. As such, hydrogels have emerged as a promising platform for diabetic wound care 12,13. Recently, they have attracted growing interest for their anti-inflammatory, antimicrobial, antioxidant, angiogenic, stimulus-responsive, and wound-monitoring properties 8,14-16.

Diabetic wounds are hallmarked by chronic inflammation and abnormal cellular phenotypes 2. Unlike normal wounds, they often exhibit delayed or impaired healing due to factors such as hypoxia, elevated matrix metalloproteinases (MMPs) activity, and immune dysfunction 17,18. A dysregulated immune microenvironment is now recognized as a major barrier to effective healing 12,19,20. Immune cells such as macrophages, neutrophils, and T lymphocytes normally orchestrate tissue repair through pathogen clearance, cytokine secretion, and resolution of inflammation. In diabetes, however, macrophage polarization skews toward the pro-inflammatory M1 phenotype, neutrophils form excessive extracellular traps, and T cell function is impaired—collectively sustaining inflammation and hindering repair 21-23.

Given these immune abnormalities, strategies to restore a balanced immune environment are urgently needed. Hydrogels, with their tunable physicochemical properties and capacity for controlled delivery, represent an ideal platform for implementing such immunomodulatory strategies. Beyond serving as moisture-retaining dressings, hydrogels can be engineered to encapsulate and release immunomodulatory agents—including cells, extracellular vesicles (EVs), cytokines, antibodies, and small molecules—in a sustained or stimuli-responsive fashion, directly targeting the immunological deficits of diabetic wounds 13. By linking material design to the regulation of immune responses, hydrogel platforms offer a unique opportunity to integrate wound protection with active immunotherapy.

In addition to their physicochemical tunability, the degradation behavior of hydrogels is a pivotal determinant of sustained release, yet it has often been overlooked in the context of diabetic wound healing. Hydrogels degrade via hydrolytic, enzymatic, or oxidative mechanisms, with MMP and elevated reactive oxygen species (ROS) in diabetic wounds profoundly influencing their stability. Such degradation not only governs mesh size and porosity but also directly controls the kinetics and duration of therapeutic release. For instance, MMP-responsive hydrogels have been engineered to achieve on-demand growth factor delivery, synchronizing cargo release with periods of high protease activity in chronic wounds 24. However, uncontrolled degradation in the hyperinflammatory setting may exhaust payloads too early, while overly stable hydrogels may fail to release sufficient agents when needed. Therefore, future hydrogel platforms for diabetic wound immunomodulation should incorporate tunable, environment-responsive degradation mechanisms, such as MMP-sensitive linkers or ROS-degradable crosslinks, and be assessed under standardized in-vitro models that mimic diabetic wound biochemistry (e.g., acidic pH, high ROS, and elevated protease levels) 25. Taken together, degradation dynamics must be integrated into the broader design of immunomodulatory hydrogel platforms.

**Figure 1:**
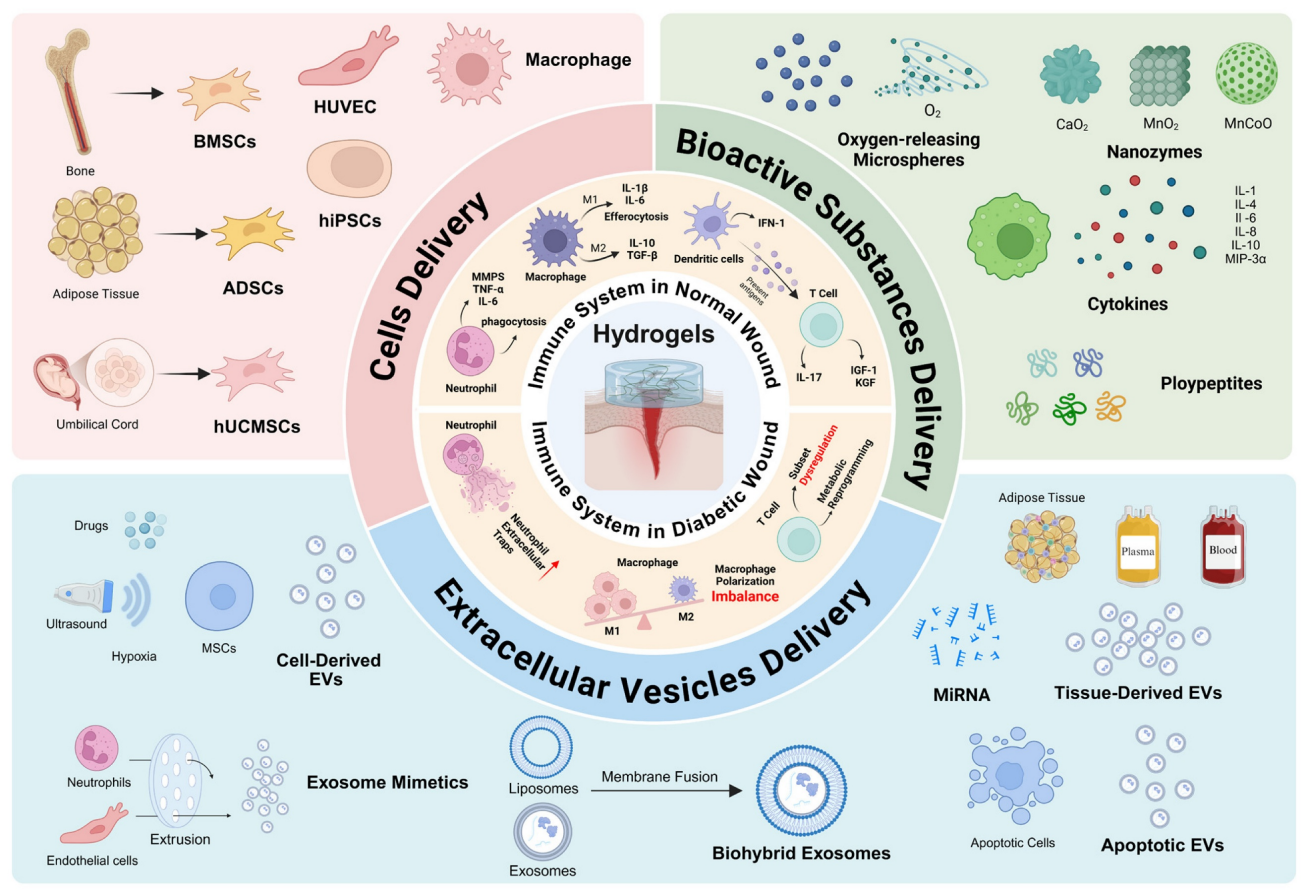
** Immunomodulatory strategies of hydrogels for the treatment of diabetic wounds.** Hydrogels act as versatile carriers to deliver (i) cells, including MSCs, macrophages, and iPSC-derived lineages; (ii) EVs from cellular, tissue, or other sources; and (iii) bioactive substances including oxygen-releasing microspheres, nanozymes, cytokines, peptides, and growth factors. These hydrogel-based platforms reshape the diabetic wound immune microenvironment by promoting macrophage polarization toward the M2 phenotype, reducing NETs, modulating T-cell responses, alleviating oxidative stress, and enhancing angiogenesis. Collectively, these strategies restore immune balance and accelerate tissue repair. MSC, mesenchymal stem cell; iPSC, induced pluripotent stem cell; EV, extracellular vesicle; NET, neutrophil extracellular trap. Created with https://BioRender.com.

Despite extensive research on the immunomodulatory roles of hydrogels, recent systematic reviews on hydrogel-based delivery systems for immune regulation in diabetic wound treatment remain limited. This review offers a comprehensive synthesis of recent advances in the development of immunomodulatory hydrogel platforms, with a particular focus on their application in diabetic wound care. It first delineates the immunological mechanisms underlying normal wound repair and highlight the distinct immune impairments associated with diabetes. We then discuss emerging strategies that incorporate hydrogels with cells, EVs, and bioactive substances to modulate immune responses in diabetic wounds (**Figure 1**). The discussion then shifts to current technological progress, existing hurdles, and future perspectives in engineering immunomodulatory hydrogels for diabetic wound therapy.

## 2. The Role of the Immune System in Wound Healing

The healing of skin wounds typically proceeds through four overlapping but sequential phases: hemostasis, inflammation, proliferation, and remodeling. The hemostatic phase is initiated by vasoconstriction and platelet activation, followed by the inflammatory phase, which is mediated primarily by neutrophils and monocytes/macrophages. During the proliferative phase, granulation tissue forms, and neovascularization occurs. The remodeling phase then involves collagen shift and extracellular matrix (ECM) remodeling. Immune cells and the bioactive factors they secrete play indispensable roles throughout all these phases.

### 2.1. Hemostatic Phase

Immediately following injury, vascular disruption leads to hemorrhage. In response, the body activates a series of coagulation mechanisms, including reactive vasoconstriction, primary coagulation, and secondary coagulation. During primary coagulation, activated platelets release growth factors and cytokines that recruit neutrophils, macrophages, and other immune cells 3,20,26. The fibrin clot formed during secondary coagulation not only halts bleeding but also provides a temporary matrix for immune cell infiltration and coagulation 7,22,27.

### 2.2. Inflammatory Phase

In the inflammatory phase, a variety of immune cells—particularly neutrophils and macrophages—coordinate the regulation of the wound immune microenvironment. Neutrophils serve as early responders rapidly recruited to the injury site upon stimulation by damage-associated molecular patterns (DAMPs), interleukin-8 (IL-8), and other chemotactic cues. They eliminate pathogens and cellular debris via phagocytosis, protease release, neutrophil extracellular traps (NETs) formation, and secretion of pro-inflammatory cytokines such as tumor necrosis factor-alpha (TNF-α) and interleukin-6 (IL-6), which amplify immune cell recruitment 3,28,29.

Subsequently, monocytes migrate from the circulation and differentiate into macrophages, primarily of the M1 phenotype in the early stages 7,22. Macrophages are also activated by DAMPs to release pro-inflammatory mediators, which further recruit neutrophils and monocytes. In this phase, macrophages play a pivotal role in clearing pathogens and cellular debris 20. Macrophages exhibit phenotypic plasticity and can be broadly classified into pro-inflammatory M1 and anti-inflammatory M2 subtypes, with M2 further subtyped into M2a, M2b, and M2c based on their functional profiles 22,28-30.

After neutrophils complete their clearance tasks, they undergo programmed cell death and are phagocytosed by M1 macrophages through efferocytosis, which triggers the reprogramming of macrophages toward the M2 phenotype 29,31. This transition is essential for shifting the immune microenvironment from a suppressive to a reparative state.

### 2.3. Proliferative and Remodeling Phase

The M1-to-M2 transition of macrophages marks the beginning of the proliferative and remodeling phases. During this period, macrophages support neovascularization, stimulate fibroblast differentiation into myofibroblasts, and may even transdifferentiate into fibroblast-like cells 20,28,29,31. In the early proliferative phase, M2a macrophages become predominant, secreting pro-fibrotic cytokines and depositing ECM components. However, prolonged retention of M2a macrophages can exacerbate scar formation 30-32. During the remodeling phase, the macrophage population declines and becomes dominated by M2c macrophages, which release MMPs that degrade excessive ECM and contribute to matrix remodeling 22,30-32.

In addition to macrophages and neutrophils, other immune and non-immune cells are involved in coordinating the immune response during wound healing 31,33,34. T cells and mast cells contribute by recruiting immune cells and regulating fibrosis and angiogenesis 35,36. Dendritic cells secrete type I interferons (IFNs) and promote transforming growth factor-beta 1 (TGF-β1) production, thereby supporting granulation tissue formation and vascular regeneration during the proliferative phase 37. Adipocytes also play a role, especially in the early phase of healing. In Drosophila, adipose-like cells migrate directionally to the wound site, assisting macrophages in clearing debris 38. In humans, subcutaneous adipocytes secrete lipocalin and leptin to modulate macrophage polarization. Additionally, lipolysis-derived fatty acids enhance macrophage recruitment in the early wound healing stage 34,39. Fibroblasts, which exhibit both pro-inflammatory and pro-fibrotic phenotypes, actively regulate immune cell recruitment and function throughout the wound healing process 33,34,40.

## 3. Immune Dysregulation in Diabetic Wound Healing

The impaired healing of diabetic wounds is closely associated with persistent inflammation, which stems from various immune abnormalities, including the excessive accumulation of neutrophils, macrophage phenotypic imbalances, and dysregulation of T-cell ratios and functions. Therefore, identifying and addressing these immune dysfunctions is critical for the effective treatment of diabetic wounds.

### 3.1. Macrophage Dysfunction in Diabetic Wounds

Macrophage dysfunction is a major contributor to the delayed healing observed in diabetic wounds. The hyperglycemic microenvironment profoundly affects macrophage polarization, leading to a functional imbalance that disrupts the wound healing process. A hallmark of this dysfunction is the skewed polarization toward the proinflammatory M1 phenotype, with a concomitant reduction in the reparative M2 phenotype. M1 macrophages continuously secrete proinflammatory cytokines such as TNF-α and interleukin-1β (IL-1β), which intensify inflammation and stimulate the production of MMPs. These enzymes degrade the ECM, exacerbating wound chronicity. Meanwhile, the deficiency of M2 macrophages impairs the resolution of inflammation and tissue regeneration 41,42.

In the diabetic microenvironment, advanced glycation end products (AGEs) interact with macrophage receptors and activate the NF-κB signaling pathway. This activation enhances the proinflammatory activity of M1 macrophages, suppresses M2 macrophage functions, and increases the release of inflammatory mediators 29. AGEs also significantly impair macrophage phagocytosis, particularly the clearance of apoptotic cells and pathogens. This results in the accumulation of cellular debris and prolongs the inflammatory phase. Moreover, impaired efferocytosis hampers the macrophage phenotypic switch from M1 to M2 29,43-45.

Notably, local insulin application has been shown to promote the M1-to-M2 phenotypic transition in diabetic wounds. Insulin signaling abnormalities, a hallmark of the diabetic state, have also been identified as key drivers of macrophage dysfunction 46. Additionally, the diabetic environment disrupts macrophage interactions with other immune cells. For instance, diminished efferocytosis allows neutrophil accumulation and excessive ROS production, which can prematurely impair fibroblast functions 20,43-45.

In summary, macrophage dysfunction in diabetic wounds is characterized by phenotypic imbalance, impaired phagocytosis, excessive proinflammatory cytokine production, and disrupted cellular crosstalk. Restoring macrophage function, especially promoting their polarization toward the M2 phenotype, has become a central strategy in diabetic wound therapy. For example, PPAR-γ agonists have been shown to enhance neutrophil clearance and promote M2 polarization, thereby facilitating wound healing 47.

### 3.2. Neutrophil Dysfunction in Diabetic Wounds

Neutrophil dysfunction is another important factor contributing to poor healing in diabetic wounds. Under diabetic conditions, excessive neutrophil accumulation and activation lead to increased oxidative stress and overproduction of ROS, which in turn activate proteases that damage the ECM and aggravate local inflammation 20,31. Moreover, proteases such as neutrophil elastase degrade critical growth factors necessary for tissue repair, including platelet-derived growth factor (PDGF) and TGF-β1 20,21.

The dysregulation of neutrophil apoptosis also contributes to persistent inflammation. Apoptotic neutrophils are frequently observed in diabetic wounds, and AGEs have been shown to impair their clearance by macrophages 43,48. Recently, attention has focused on NETs, which are web-like structures composed of chromatin and antimicrobial proteins that trap and kill pathogens 49. In diabetes, increased activity of enzymes such as peptidyl arginine deiminase 4 (PAD4), neutrophil elastase, and protein kinase C leads to excessive NET formation. This exacerbates inflammation, damages the ECM, and impairs the wound healing environment 50.

Mechanistically, NETs activate the NOD-like receptor family pyrin domain containing 3 (NLRP3) inflammasome and promote IL-1β secretion through Toll-like receptor (TLR) and NF-κB pathways 49. They also inhibit angiogenesis via dysregulation of the Hippo-YAP signaling pathway and interfere with fibroblast function, reducing collagen and ECM protein synthesis 49,51. The overproduction of NETs thus hinders both vascularization and fibroblast-mediated tissue repair. Consequently, the use of NET inhibitors or other NET-targeting strategies has emerged as a promising approach to improving the immune microenvironment of diabetic wounds 49,52-54.

### 3.3. T Cell Dysfunction in Diabetic Wounds

T cell dysfunction in diabetic wounds represents a complex interplay of metabolic reprogramming and altered subset dynamics, driven primarily by hyperglycemia. Elevated glucose levels have been shown to induce mitochondrial dysfunction in CD4+ T cells, resulting in increased oxidative stress and lipid accumulation. This metabolic stress promotes lipid peroxidation (LPO) and STAT4 carbonylation, ultimately impairing T cell function 55.

In addition, T cell subset imbalance is a prominent feature of diabetic wound immunopathology. The diabetic milieu promotes the expansion of effector T cells in circulation while reducing the diversity of the TCR-β pool. These effector T cells secrete high levels of IFN-γ and TNF-α, exacerbating the inflammatory response 21,56. Meanwhile, regulatory T cells (Tregs), which are essential for immune tolerance and inflammation resolution, are reduced in number or functionally impaired in diabetic wounds. In T2D, hyperinsulinemia has been found to further suppress Treg functions, amplifying immune dysregulation 57-59.

Insulin-like growth factor 1 (IGF-1) plays a crucial role in wound healing by promoting cell proliferation, migration, collagen synthesis, and angiogenesis. It also modulates immune responses to facilitate repair 60. However, IGF-1 signaling is impaired in diabetic patients, and T cells from diabetic wounds often lack the ability to produce IGF-1, contributing to delayed wound healing and immune dysfunction 21,56,61.

### 3.4. Adipocyte and Fibroblast Dysfunction in Diabetic Wounds

Although adipocytes and fibroblasts are not immune cells, they play pivotal roles in the immunomodulation of diabetic wounds. In diabetic patients, adipocytes exhibit increased secretion of pro-inflammatory factors, which promote macrophage recruitment while inhibiting the polarization toward M2 phenotypes. Additionally, diabetic fibroblasts undergo premature senescence and develop a senescence-associated secretory phenotype (SASP), characterized by elevated expression of pro-inflammatory cytokines, chemokines, and MMPs. These factors collectively contribute to impaired wound healing and tissue regeneration 34.

### 3.5. Crosstalk Among Immune Cells in Diabetic Wounds

Beyond individual cellular dysfunctions, diabetic wounds are sustained by complex crosstalk among macrophages, neutrophils, and T cells 28,31. Pro-inflammatory M1 macrophages secrete IL-1β and TNF-α, which enhance neutrophil recruitment and survival 21,22,32. Neutrophils, in turn, release excessive NETs that activate the macrophage NLRP3 inflammasome, amplifying inflammatory signaling 21. Macrophage-derived IL-12 and IL-23 further drive Th1/Th17 polarization, while Th17-derived IL-17 reinforces neutrophil infiltration 22,32. Conversely, Tregs release IL-10 and TGF-β to restrain M1 macrophage activity and restore immune balance, but their numbers and function are markedly reduced in diabetes 27. These multidirectional interactions indicate that chronic inflammation in diabetic wounds arises not from isolated defects but from interconnected networks of immune dysregulation, underscoring the need for therapeutic strategies that intervene at multiple cellular nodes simultaneously.

### 3.6. Potential Therapeutic Targets

Although hyperglycemia-induced immune dysfunction involves multiple cellular populations, recent evidence suggests that certain targets may be more tractable than others. Among these, macrophage polarization has emerged as the most promising therapeutic node. Driving macrophages from a pro-inflammatory M1 phenotype toward a reparative M2 phenotype not only suppresses excessive cytokine release but also promotes angiogenesis and matrix remodeling, thereby playing a central role in coordinating inflammation resolution and tissue repair. In addition, neutrophil hyperactivation and excessive formation of NETs have increasingly been recognized as pathological hallmarks of diabetic wounds, contributing to persistent inflammation, proteolytic tissue damage, and delayed re-epithelialization. By contrast, strategies aimed at harnessing T cells or non-immune cell types for immunomodulation remain in earlier stages, with limited available interventions and insufficient preclinical validation. Taken together, targeting macrophage polarization and NET clearance provides two promising avenues for restoring immune homeostasis and accelerating repair in diabetic wounds.

## 4. Application of the Immunomodulatory Hydrogel Delivery System

Hydrogels have garnered significant attention in diabetic wound therapy owing to their outstanding biocompatibility, customizable mechanical properties, and superior capacity for sustained delivery. These attributes make them ideal platforms for encapsulating and delivering immunomodulatory agents. Although hydrogels themselves can influence immune responses through their intrinsic physicochemical properties—such as stiffness, porosity, degradation kinetics, and composition—the magnitude of these effects is generally modest compared to the potent bioactivity of the encapsulated cargo. Therefore, this review primarily focuses on the immunomodulatory effects mediated by hydrogel-delivered agents, rather than the intrinsic immunomodulatory capacity of the hydrogels alone. Hydrogels can be engineered to achieve controlled release of therapeutic cargos, such as cells, EVs, anti-inflammatory drugs, and other bioactive substances, thereby modulating the immune microenvironment and enhancing tissue repair. This section highlights on the application of immunomodulatory hydrogels in diabetic wound healing.

### 4.1. Cells

Cell-based therapies, particularly those involving mesenchymal stem cells (MSCs), have shown considerable potential for diabetic wound repair. MSCs not only possess self-renewal capabilities but also differentiate into fibroblasts, endothelial cells, and other regenerative cells. Importantly, they exhibit potent immunomodulatory functions by anti-inflammatory mediators, growth factors, and other paracrine signals 12,62. During tissue repair, MSCs have been shown to facilitate the macrophage phenotypic switching of from M1 to M2 through the secretion of factors like TGF-β, IL-6 63,64. Moreover, MSCs can suppress TNF-α secretion from M1 macrophages and promote a shift in T cell from the pro-inflammatory Th1 to the anti-inflammatory Th2 subtype 65,66. These findings underscore the therapeutic relevance of MSCs in ameliorating chronic inflammation in diabetic wounds, providing a scientific rationale for their use in clinical treatment (**Table 1**).

#### 4.1.1. Bone Marrow Mesenchymal Stem Cell

The safety and efficacy of bone marrow mesenchymal stem cells (BMSCs) have been extensively validated in clinical settings. Chen *et al.* developed a BMSC-based hydrogel delivery system that enhanced fibroblast, endothelial cell, and keratinocyte functions via the secretion of TGF-β1 and basic fibroblast growth factor (bFGF) 67. The hydrogel also suppressed M1 macrophage activity, thereby ameliorating the inflammatory milieu and accelerating wound healing. However, the study did not directly demonstrate BMSC-mediated immunomodulation. In contrast, Bai *et al.* utilized a self-healing hydrogel to deliver BMSCs and reported not only suppression of M1 macrophage activation but also promotion of M2 polarization 68. Notably, this enhanced M2 polarization was mechanistically linked to the presence of BMSCs, suggesting that their immunomodulatory potential may be context-dependent and remains a subject of ongoing debate.

#### 4.1.2. Adipose-derived Mesenchymal Stem Cell

Adipose-derived mesenchymal stem cells (ADSCs) have garnered increasing interest for cutaneous regeneration owing to their ease of isolation, robust differentiation capacity, and strong immunomodulatory effects 78. Shi *et al.* demonstrated that ADSCs can enhance macrophage polarization toward an M2-like profile 79. However, the survival and functionality of ADSCs in the hostile diabetic wound environment remain major challenges. To address this, Dong *et al.* constructed an injectable hydrogel that extended ADSC survival in diabetic wounds for up to 14 days, while enhancing angiogenesis and suppressing inflammation 70. Similarly, Xia *et al.* incorporated curcumin into the hydrogel matrix to reduce ADSC apoptosis, thereby improving cell viability and promoting wound repair 71.

#### 4.1.3. Human Umbilical Cord-derived Mesenchymal Stem Cell

Comparable to BMSCs and ADSCs, human umbilical cord-derived mesenchymal stem cells (hUCMSCs) exhibit robust immunoregulatory functions and multipotency. They offer distinct advantages, such as low immunogenicity, non-invasive collection procedures, and minimal ethical concerns. Through mechanisms including multilineage differentiation, paracrine signaling, and anti-inflammatory activity, hUCMSCs significantly contribute to diabetic wound repair 80. Encapsulation of hUCMSCs in hydrogels enhances their therapeutic efficacy, downregulates pro-inflammatory mediators, while concurrently promoting angiogenesis 72-74.

#### 4.1.4. Macrophage

Beyond stem cell delivery, recent investigations have also explored macrophage transplantation via hydrogel scaffolds as a strategy to expedite diabetic wound repair. Given the pivotal role of macrophages in shaping the immune milieu of chronic wounds, targeted delivery of these cells represents a promising immunotherapeutic avenue 76,81,82. For example, Georgios *et al.* loaded M0, M1, M2a, and M2c macrophages, along with their secretome, into an alginate dressing, and found that macrophages, regardless of their polarization state, can improve wound healing, while the delivered M2 macrophages were able to maintain or promote similar polarization states 76.

#### 4.1.5. Induced Pluripotent Stem Cell-Derived Cells

Human induced pluripotent stem cells (hiPSCs) represent a renewable and patient-compatible source for multiple cell lineages, including endothelial cells (hiPSC-ECs), smooth muscle cells (hiPSC-SMCs), fibroblasts (hiPSC-FBs), and mesenchymal stem cells (hiPSC-MSCs) 83. In addition to their regenerative potential, hiPSC-derived cells have shown emerging immunomodulatory activity, such as suppression of pro-inflammatory cytokine production and promotion of macrophage polarization. These dual properties address two critical barriers to diabetic wound healing: chronic inflammation and ischemia 84,85.

##### 4.1.5.1. hiPSC-ECs

Among hiPSC-derived lineages, hiPSC-ECs are the most extensively studied in diabetic wounds, where they have been shown to accelerate angiogenesis and restore endothelial function 86. For instance, nanovesicles derived from hiPSC-ECs and loaded with dapagliflozin enhanced HIF-1α/VEGFA signaling, promoted neovascularization, and improved wound closure, suggesting a paracrine or gene-regulatory mechanism rather than direct engraftment 87. While most work highlights vascular repair, endothelial cells are known to crosstalk with innate immune populations, including neutrophils and macrophages, implying that future hiPSC-EC strategies could exploit a vascular-immune axis to integrate angiogenesis with immunomodulation.

##### 4.1.5.2. hiPSC-FBs

hiPSC-FBs recapitulate the transcriptional profiles of primary dermal fibroblasts and provide a scalable source for individualized dermal reconstruction. Although their direct immunoregulatory role in diabetic wounds is less defined, evidence from cutaneous and mucosal repair demonstrates that certain fibroblast subsets can modulate immune responses and support regeneration 88. Moreover, iPSC-derived extracellular matrix has been shown to reshape the wound environment, indicating that fibroblasts may contribute to both structural and immunological aspects of repair 89.

##### 4.1.5.3. hiPSC-SMCs

hiPSC-SMCs support vessel maturation and secrete pro-angiogenic mediators. Importantly, in diabetic wound models, the delivery of hiPSC-SMCs not only accelerated healing but also increased the prevalence of M2 macrophages, suggesting that their therapeutic effect extends beyond angiogenesis to active immunomodulation. This positions hiPSC-SMCs as a lineage capable of bridging vascular stabilization with immune rebalancing 77.

##### 4.1.5.4. hiPSC-MSCs

hiPSC-derived MSCs combine the regenerative and immunomodulatory features of conventional MSCs with the scalability and autologous compatibility of hiPSCs. While direct data on their use in diabetic wounds remains limited, accumulating evidence indicates that hiPSC-MSCs, particularly through extracellular vesicles, can suppress macrophage-derived pro-inflammatory cytokines, enhance angiogenesis, and accelerate wound repair. These paracrine effects underscore their promise as an immunoregulatory therapy, although head-to-head comparisons with conventional MSCs are still needed 90.

##### 4.1.5.5. hiPSC-Derived Immune Cells

As an emerging avenue, standardized platforms now allow mid-scale production of functionally mature hiPSC-derived immune cells. Notably, hiPSC-macrophages exhibit transcriptional and chromatin landscapes comparable to peripheral blood-derived macrophages during M1/M2 polarization. This raises the possibility of engineering hiPSC-derived macrophages for therapeutic use, such as promoting M2 polarization or enhancing the clearance of NETs. Incorporating such cells into hydrogel systems could represent a next-generation strategy for direct immune cell-based therapy in diabetic wounds 91,92.

Overall, hiPSC-ECs and hiPSC-SMCs primarily act through vascular regeneration, with hiPSC-SMCs already showing immunomodulatory effects via M2 polarization. hiPSC-FBs and their ECM remodeling capacity may further shape the immune milieu, while hiPSC-MSCs and their EVs provide paracrine immunomodulation. Future studies should incorporate immune endpoints—such as macrophage polarization, T cell balance, and cytokine profiles—to establish immunoregulatory efficacy. Nonetheless, several challenges remain: (i) safety and phenotypic stability, given the risks of residual pluripotency and phenotypic drift; (ii) survival and functional persistence in the hostile diabetic wound microenvironment, which necessitate protective hydrogel formulations with antioxidant or protease-adaptive properties; and (iii) scalability and GMP-standardized differentiation, as batch-to-batch variation in iPSC-derived products and hydrogel manufacturing remain bottlenecks for translation. Addressing these limitations will be essential to realize the full potential of hiPSC-derived cells as dual regenerative and immunomodulatory agents in hydrogel-based diabetic wound therapy.

#### 4.1.6. Strategies to Improve Cell Delivery Efficiency

Despite the promising results of cell-based therapies, several obstacles remain, particularly the limited survival and activity of transplanted cells in the inflammatory, hypoxic, and hyperglycemic wound microenvironment. These conditions attenuate cytokine secretion and impair therapeutic outcomes 93. While hydrogel encapsulation improves cell viability and retention, additional strategies are required to optimize therapeutic benefits.

Pre-conditioning cells before transplantation has shown the potential to improve survival and function 94. For example, Anisa *et al.*  demonstrated that FGF21 pretreatment enhanced BMSC viability in a hyperglycemic context, thereby promoting wound healing 69. Similarly, melatonin preconditioning was reported to boost the immunomodulatory effects of hUCMSCs, particularly in regulating macrophage polarization 95. Genetic engineering of therapeutic cells is another emerging approach. Modifying cellular gene expression not only enhances cell survival but also tailors their secretory profiles for targeted therapy 96. Huang *et al.* developed a hydrogel platform encapsulating human umbilical vein endothelial cells (HUVECs) overexpressing VEGF165, which mitigated mitochondrial damage and downregulated multiple pro-inflammatory cytokines, ultimately improving the immune environment and facilitating diabetic wound healing 75.

### 4.2. Extracellular Vesicles

Despite extensive efforts by researchers, ranging from the development of hydrogel systems, preconditioned cells, and gene editing strategies to the exploration of alternative cell types beyond stem cells, cell-based therapies for diabetic wounds remain suboptimal.

The notable regenerative potential of the cellular secretome, particularly that of stem cells, has redirected attention toward the use of EVs for diabetic wound healing 76. Compared to their cellular origins, EVs offer lower immunogenicity, improved safety, greater stability, and enhanced drug-loading capacity, granting them a competitive edge in clinical applications, especially in tissue repair. Furthermore, mounting evidence indicates that the immunomodulatory capacity of MSCs is predominantly mediated by their secreted EVs 97,98.

However, the rapid clearance of EVs when administered alone remains a major limitation. To address this, hydrogels have gained widespread application as sustained-release matrices to improve their retention (**Table 2**). The relationship between hydrogels and EVs is mutually beneficial: hydrogels protect EVs from immune clearance and enable their sustained release, while EVs enhance the immunomodulatory properties of hydrogels, suppress inflammation, and promote cellular proliferation and migration. Besides, EVs can be engineered as carriers for therapeutic molecules involved in wound repair, offering a versatile strategy for enhanced healing outcomes 99,100.

#### 4.2.1. Cell-Derived Extracellular Vesicles

##### 4.2.1.1. BMSCs-Derived Extracellular Vesicles

BMSCs-derived EVs (BMSC-EVs) have been shown to inhibit M1 macrophage polarization and foster M2 polarization. These vesicles suppress the NF-κB/p65 signaling pathway and downregulate pro-inflammatory cytokines 62,117,118. Additionally, BMSC-EVs support wound healing by enhancing cell proliferation, migration, and angiogenesis 62,119. Wang *et al.* engineered a BMSC-EV-laden hydrogel, which modulated IL-6 expression in diabetic wounds 101. Moreover, Geng *et al.* incorporated BMSC-EVs into a chitosan hydrogel to shift macrophage phenotype from M1 to M2, thereby reshaping the local immune microenvironment 102.

##### 4.2.1.2. ADSCs-derived Extracellular Vesicles

**Figure 2:**
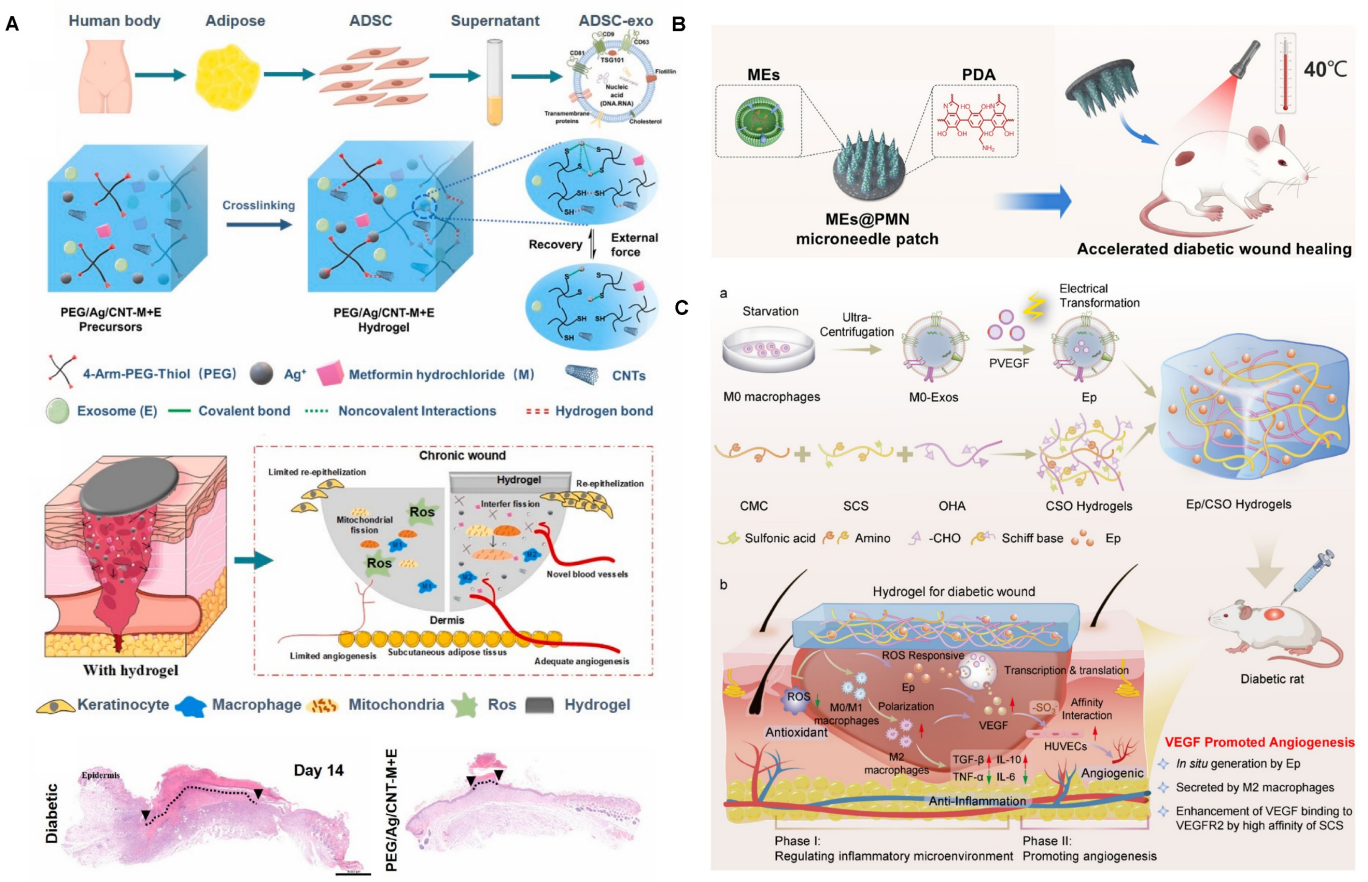
**Immunomodulatory hydrogel systems for delivering cell-derived EVs.** (A) Preparation and application of the PEG/Ag/CNT-M+E hydrogel for diabetic wounds. Adapted with permission from 103. Copyright 2023, Elsevier. (B) Immunomodulation of the M2 macrophage-derived EV-encapsulated microneedles with PDA (MEs@PMN) for diabetic wound healing. Adapted with permission from 108. Copyright 2023, Elsevier. (C) Fabrication and therapeutic mechanisms of regulating the inflammatory microenvironment and promoting angiogenesis in diabetic wound healing by Ep/CSO hydrogels. Adapted with permission from 109. Copyright 2025, American Chemical Society.

Adipose-derived stem cell EVs (ADSC-EVs) also exhibit potent immunomodulatory effects 119. Zhang *et al.* co-loaded ADSC-EVs and metformin into an injectable conductive hydrogel that suppressed vascular inflammation and reduced IL-6 and TNF-α secretion (**Figure 2A**). The hydrogel also mitigated mitochondrial fission and ROS generation in hyperglycemic HUVECs, thereby promoting angiogenesis 103. Similarly, Song *et al.* developed an ECM-based hydrogel loaded with ADSC-EVs, which significantly downregulated IL-6 and TNF-α levels at the wound site 104. To achieve precision delivery, Jiang *et al.* employed MMP-responsive smart hydrogels to release ADSC-EVs upon MMP-2 stimulation, reactivating the suppressed AKT signaling pathway in diabetic wounds and promoting cell proliferation and migration 120.

##### 4.2.1.3. hUCMSCs-derived Extracellular Vesicles

Human umbilical cord MSC-derived EVs (hUCMSC-EVs) accelerate diabetic wound closure by enhancing angiogenesis and promoting fibroblast proliferation and migration 121,122. Their immunomodulatory functions have also attracted increasing attention. For instance, hUCMSC-EVs suppress monocyte chemoattractant protein-1 (MCP-1) in retinal injury, thereby attenuating inflammation 123. Moreover, they reduce the expression of IL-6, TNF-α, iNOS, IL-1β, IL-7, and other pro-inflammatory cytokines; ameliorate insulin resistance; enhance glucose metabolism; and inhibit pancreatic β cell apoptosis under hyperglycemic conditions, offering promise for type 2 diabetes therapy 124,125. Injecting hUCMSC-EVs into type 1 diabetic mice via the tail vein improved insulin secretion and lowered the mice's blood sugar levels. At the same time, the wounds of the mice were treated with a hydrogel with anti-inflammatory effects. The synergistic effect of the two further improved the wound healing of type 1 diabetic mice 126. Co-loading hUCMSC-EVs with a histone deacetylase 7 (HDAC7)-derived peptide into alginate hydrogels significantly reduced pro-inflammatory cytokine production from macrophages in diabetic wounds 106.

##### 4.2.1.4. Other cell-Derived Extracellular Vesicles

Beyond the common stem cell sources, other cell types also yield EVs with immunomodulatory potential. Foreskin-derived MSCs (FSMSCs), which are ethically favorable and possess stronger proliferative and immunoregulatory properties than hUCMSCs, are one such example 107,127. Xu *et al.* isolated FSMSCs and embedded FSMSC EVs (FM-EVs) into a polyvinylpyrrolidone/silicotungstic acid-based hydrogel. These FM-EVs promoted angiogenesis and induced M2 macrophage polarization via let-7b-5p, thereby accelerating diabetic wound healing 107.

Immune cell-derived EVs also hold promise. For example, M2 macrophage-derived EVs (M2-EVs) promote M2 polarization through the delivery of CCL22, CCL24, and MFG-E8 128,129. Zeng *et al.* co-delivered M2-EVs and polydopamine (PDA) nanoparticles via hydrogel microneedles 108. These systems not only enhanced M2 polarization but also boosted angiogenesis through the photothermal effects of PDA (**Figure 2B**). In addition, M0 macrophage-derived EVs embedded in immunomodulatory hydrogels have been shown to attenuate inflammation, and engineered loading of VEGF plasmids into these EVs further enhanced angiogenesis, demonstrating synergistic promotion of diabetic wound repair (**Figure 2C**) 109. Tregs also secrete EVs with strong immunomodulatory potential 130. Treg-derived EVs (Treg-EVs) modulate macrophages, dendritic cells, and T cells via miRNAs, enzymes, and surface proteins 131. Cord blood-derived Treg EVs promoted M2 polarization in monocytes and decreased local inflammatory cytokines in diabetic wounds 110.

##### 4.2.1.5. Strategies for Enhanced Efficacy of Cell-Derived Extracellular Vesicles

As with cell therapies, EVs' function can be enhanced via preconditioning strategies. Environmental stimuli significantly influence the bioactivity and cargo of EVs. For example, hypoxia-preconditioned ADSC-EVs demonstrate enhanced pro-angiogenic and anti-inflammatory effects, thereby improving diabetic wound repair 132-135. Wang *et al.* loaded hypoxia-preconditioned ADSC-Evs into *in situ* formed injectable hydrogels and verified their promoting effects on the immune microenvironment and angiogenesis of diabetic wounds both *in vivo* and *in vitro* 105. Similarly, hypoxia-treated hUCMSC-EVs inhibit excessive NET formation in diabetic wounds via miR-17-5p 54. Pharmacologic interventions are also employed to modulate EVs' function. Melatonin-treated BMSC-EVs promote M2 macrophage polarization by upregulating PTEN and inhibiting AKT phosphorylation 136. However, the impact of melatonin on EV yield, cargo, and stability remains debated 136,137.

Low EV yield and technical barriers to isolation still hinder clinical translation. CHIR99021, a Wnt/β-catenin pathway agonist, has been shown to increase hUCMSC-EV yield by 1.5-fold while modulating EV-related genes involved in TGF-β and PI3K-AKT signaling, reducing inflammation at wound sites 138-140. Astragaloside IV (ASIV) also enhances EV production from endothelial progenitor cells and improves diabetic wound inflammation 141,142. Moreover, low-intensity ultrasound stimulation effectively boosts EV secretion from ADSCs 143.

#### 4.2.2. Tissue-Derived Extracellular Vesicles

Compared to cell-derived EVs, adipose and plasma-derived EVs offer a more cost-effective and scalable alternative, while retaining comparable immunomodulatory capabilities 111,113,144. Adipose tissue consists of a heterogeneous mix of adipocytes, endothelial cells, fibroblasts, immune cells, and other cell types. EVs secreted by these various cells exert distinct effects on wound healing. Notably, EVs derived from whole adipose tissue, without enzymatic removal of specific cell populations, have shown superior therapeutic efficacy in promoting wound repair 145. For instance, Ma *et al.* incorporated adipose tissue-derived EVs (AT-EVs) into an egg white-based hydrogel, leveraging the combined antioxidant and anti-inflammatory properties of both components 111. This formulation effectively reduced excess ROS levels and encouraged macrophage polarization toward a reparative M2 phenotype, thereby significantly enhancing diabetic wound healing.

Plasma-derived EVs, particularly those originating from platelets, also contribute meaningfully to tissue repair. Platelets not only play a critical role in hemostasis and coagulation but also release a range of cytokines upon activation that modulate the wound healing process 3,20,26. Studies have demonstrated that platelet-rich plasma (PRP) accelerates diabetic wound healing, and EVs isolated from PRP exhibit similar biological functions 146,147. Wang *et al.* delivered PRP-EVs to diabetic wounds via hydrogels, which not only increased the proportion of M2 macrophages but also reduced the number of neutrophils, thereby improving excessive inflammation 112.

**Figure 3:**
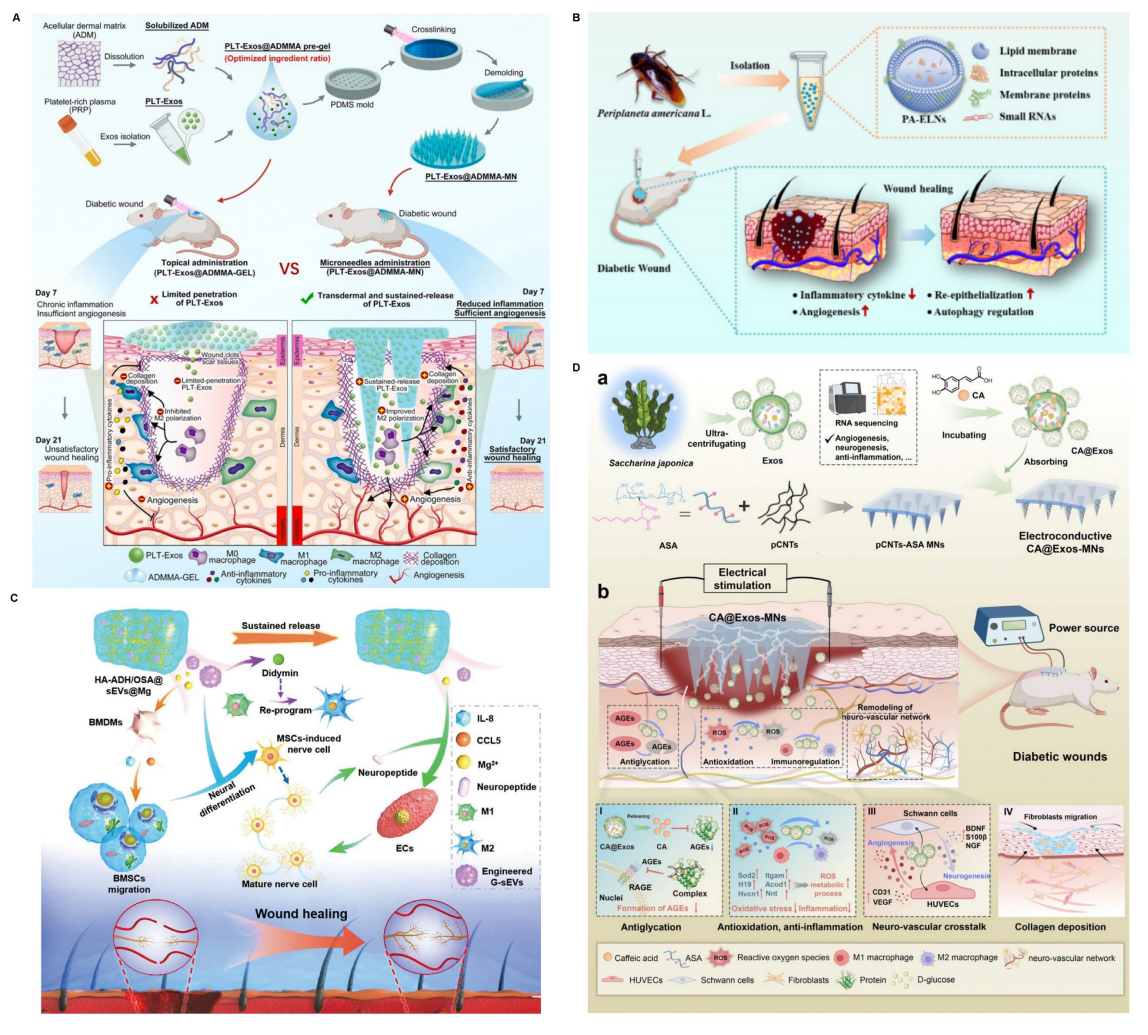
** Immunomodulatory hydrogel systems for delivering tissue-derived, plant-derived, and animals-derived EVs**. (A) Preparation, merits, and mechanisms of dissolvable MN-based wound dressing (PLT-EVs@ADMMA-MN). Adapted with permission from 114. Copyright 2024, Elsevier. (B) Isolation and application of PA-EVs. Adapted with permission from 150. Copyright 2023, Springer Nature. (C) Beneficial role of HA-ADH/OSA@Mg@sEVs hydrogel. Adapted with permission from 115. Copyright 2023, Wiley. (D) Fabrication and therapeutic mechanisms of Saccharina japonica-derived EVs-functionalized conductive microneedles. Adapted with permission from 154. Copyright 2025, Wiley.

Platelet-derived EVs (pEVs) help neutralize intracellular ROS, upregulate IL-10 expression via the TGF-β pathway, and drive macrophage polarization in favor of M2-like behavior—collectively facilitating the shift from inflammation to tissue regeneration 113. When delivered via hydrogels, pEVs not only stimulate local angiogenesis but also establish a low-inflammatory, pro-regenerative microenvironment conducive to healing (**Figure 3A**) 113,114. Additionally, EVs derived from umbilical cord blood and peripheral blood have demonstrated angiogenic and cell migration-promoting properties, though their potential to modulate immune responses in diabetic wounds remains underexplored 148,149.

#### 4.2.3. Plant-Derived and Non-Mammalian Animals-Derived Extracellular Vesicles

Although cell- and mammalian tissue-derived EVs have shown remarkable immunomodulatory efficacy in diabetic wound healing, their broader application is still hampered by low yield, manufacturing difficulties, and potential immunogenicity. To address these limitations, EVs derived from plants and non-mammalian animals have emerged as attractive alternative therapeutic candidates.

EVs derived from plant and non-mammalian animal sources offer unique biological activities and are increasingly being explored for diabetic wound therapy 115,150. For example, Liao *et al.* isolated EVs from *Periplaneta americana*, identifying a panel of miRNAs associated with Hedgehog, TGF-β, autophagy, and mTOR signaling pathways (**Figure 3B**) 150. These EVs mitigated excessive autophagy and MMP-9 overexpression, alleviating tissue damage and chronic inflammation in diabetic wounds. Similarly, EVs extracted from ginseng sap have been employed to deliver didymin—a compound known to promote M2 macrophage polarization—via Mg²⁺-incorporated hydrogels. This strategy supported wound healing by orchestrating neurogenesis, immune regulation, and angiogenesis (**Figure 3C**) 115.

Beyond ginseng-derived EVs, recent studies have explored additional plant-derived exosome-laden hydrogels. For instance, Zaffar *et al.* encapsulated rose petal-derived EVs, which possess intrinsic antibacterial properties, into an injectable hydrogel 151. This formulation effectively cleared Gram-negative bacteria from the wound surface and reduced local inflammation. However, in the diabetic context, antimicrobial activity alone is insufficient, as persistent immune imbalance—particularly the prolonged retention of M1 macrophages—remains a critical barrier. Notably, many plant-derived EVs exhibit intrinsic anti-inflammatory effects. Jin *et al.* demonstrated that lemon-derived EVs, when incorporated into hydrogels, significantly suppressed local inflammation and facilitated wound closure 152. Similarly, EVs from *Houttuynia cordata Thunb.* showed immunomodulatory potential in diabetic wound models 153. Liu *et al.* further advanced this concept by loading caffeic acid into Saccharina japonica-derived EVs and integrating them with electroconductive microneedles. This combined system promoted diabetic wound healing through multiple mechanisms, including neuroregulation, immunomodulation, angiogenesis, and inhibition of AGEs (**Figure 3D**) 154. In addition to antimicrobial and anti-inflammatory functions, certain plant-derived EVs also provide metabolic benefits specific to diabetes. Weng *et al.* reported that EVs from Momordica charantia (MC), a plant with hypoglycemic activity, not only improved glycemic control but also alleviated chronic inflammation and promoted healing in diabetic wounds 155.

Collectively, these findings highlight the translational promise of hydrogel systems incorporating plant- and non-mammalian animal-derived EVs. Owing to their low cost, scalability, and intrinsic bioactivities, such EVs represent a compelling next-generation strategy for diabetic wound immunotherapy.

#### 4.2.4. EV Mimetics

Despite the therapeutic promise of EVs, challenges related to low yield and limited modifiability constrain their broader application. EV mimetics, which mimic the structure and function of natural EVs, have emerged as a scalable and customizable alternative for drug delivery 156,157. These can be produced via methods such as extrusion, chemical induction, nitrogen cavitation, or liposome-based engineering.

**Figure 4:**
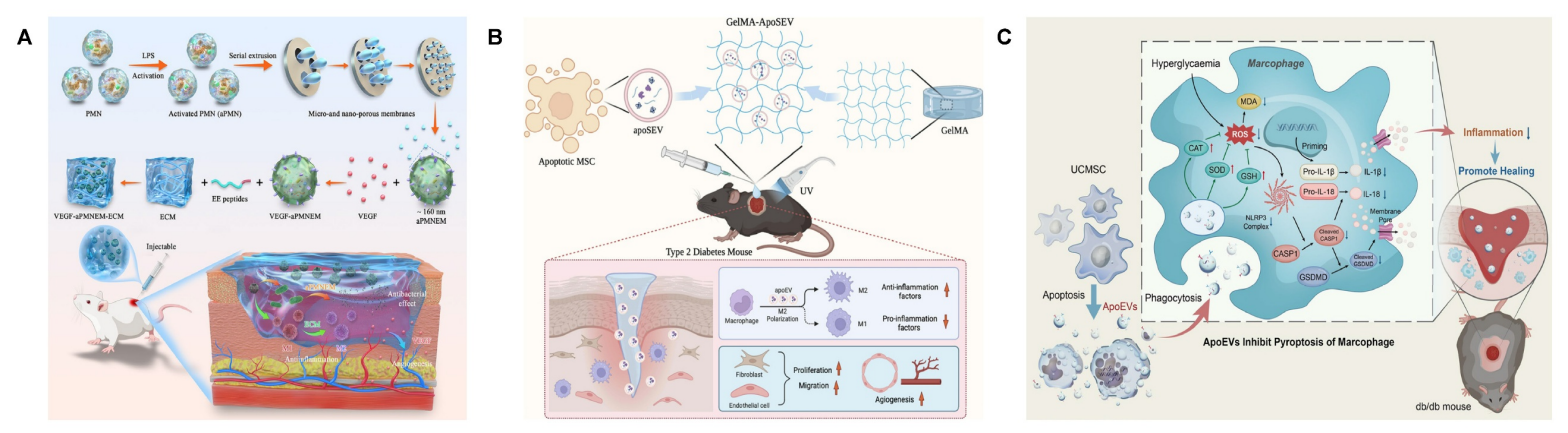
**Immunomodulatory hydrogel systems for delivering EV mimetics and apoptotic EVs.** (A) The fabrication of VEGF-aPMNEM-ECM hybrid hydrogel: preparation of activated PMN EV mimetics and vascular endothelial growth factor wrapping into aPMNEM. Adapted with permission from 158. Copyright 2023, Springer Nature. (B) MSC-derived apoptotic EVs converting macrophages towards the M2 phenotype and improving the functions of fibroblasts and endothelial cells. Adapted with permission from 116. Copyright 2020, Springer Nature. (C) The mechanisms of hUCMSC-derived apoptotic EVs inhibiting macrophage pyroptosis. Adapted with permission from 167. Copyright 2023, Springer Nature.

Yu *et al.* employed extrusion to generate mimetic EVs from polymorphonuclear neutrophils (PMNs), leveraging their inherent antimicrobial activity to deliver vascular endothelial growth factor (VEGF) and enhance healing (**Figure 4A**) 158. Similarly, Zhu *et al.* fabricated mimetic EVs from hUCMSCs via extrusion and validated their wound-healing efficacy 159. In another approach, mimetic EVs derived from induced pluripotent stem cells were used to deliver dapagliflozin (DA), a selective SGLT2 inhibitor with anti-inflammatory and angiogenic effects, to diabetic wounds 160. Such strategies open avenues for targeted delivery of immune-regulatory agents and growth factors.

Hybrid EVs, formed by fusing natural EVs with liposomes, offer an additional platform for engineering 157. For instance, hybrid vesicles generated by combining M2 macrophage EVs with M1-derived membranes exhibited anti-inflammatory activity while neutralizing pro-inflammatory cytokines through membrane receptor interactions. This technique, already tested in arthritis models, remains in early-stage exploration for diabetic wounds 161. However, the application of this method in diabetic wound management remains in the preliminary phase.

In parallel, researchers are investigating controlled-release systems to improve the spatial and temporal delivery of EVs from hydrogels. Traditional hydrogels often release EVs in an uncontrolled manner, resulting in therapeutic inefficiency. Ma *et al.* addressed this by designing a hydrogel with radial physical and biochemical gradients, facilitating both directional EV release and enhanced M2 macrophage polarization 111.

#### 4.2.5. Apoptotic Extracellular Vesicles

Apoptotic EVs, released during programmed cell death, are gaining recognition as potent mediators of tissue repair. Although apoptosis is often considered detrimental in cell therapies, recent studies highlight that apoptotic EVs modulate macrophages, endothelial cells, and fibroblasts to support healing—while the production of apoptotic EVs is greater than that of EVs under similar conditions 116,162,163. Yang *et al.* encapsulated ADSC-derived apoptotic EVs in GelMA hydrogels, observing enhanced M2 macrophage polarization and protection of fibroblasts and endothelial cells from high-glucose-induced damage (**Figure 4B**) 116.

Persistent inflammation in diabetic wounds is closely linked to hyperactivation of the NLRP3 inflammasome—a key player in innate immunity and inflammatory signaling 164. Chronic hyperglycemia exacerbates NLRP3 activity, perpetuating the release of pro-inflammatory cytokines like IL-1β and IL-18, and inhibiting angiogenesis 165,166. Wang *et al.* harvested apoptotic EVs from apoptotic hUCMSCs and showed that macrophages engulfing these vesicles exhibited reduced NLRP3 activation and pyroptosis, ultimately improving the inflammatory microenvironment (**Figure 4C**) 167. Harnessing apoptotic EVs thus represents a novel strategy for immunomodulation in diabetic wounds.

#### 4.2.6. miRNAs in Extracellular Vesicles

**Figure 5:**
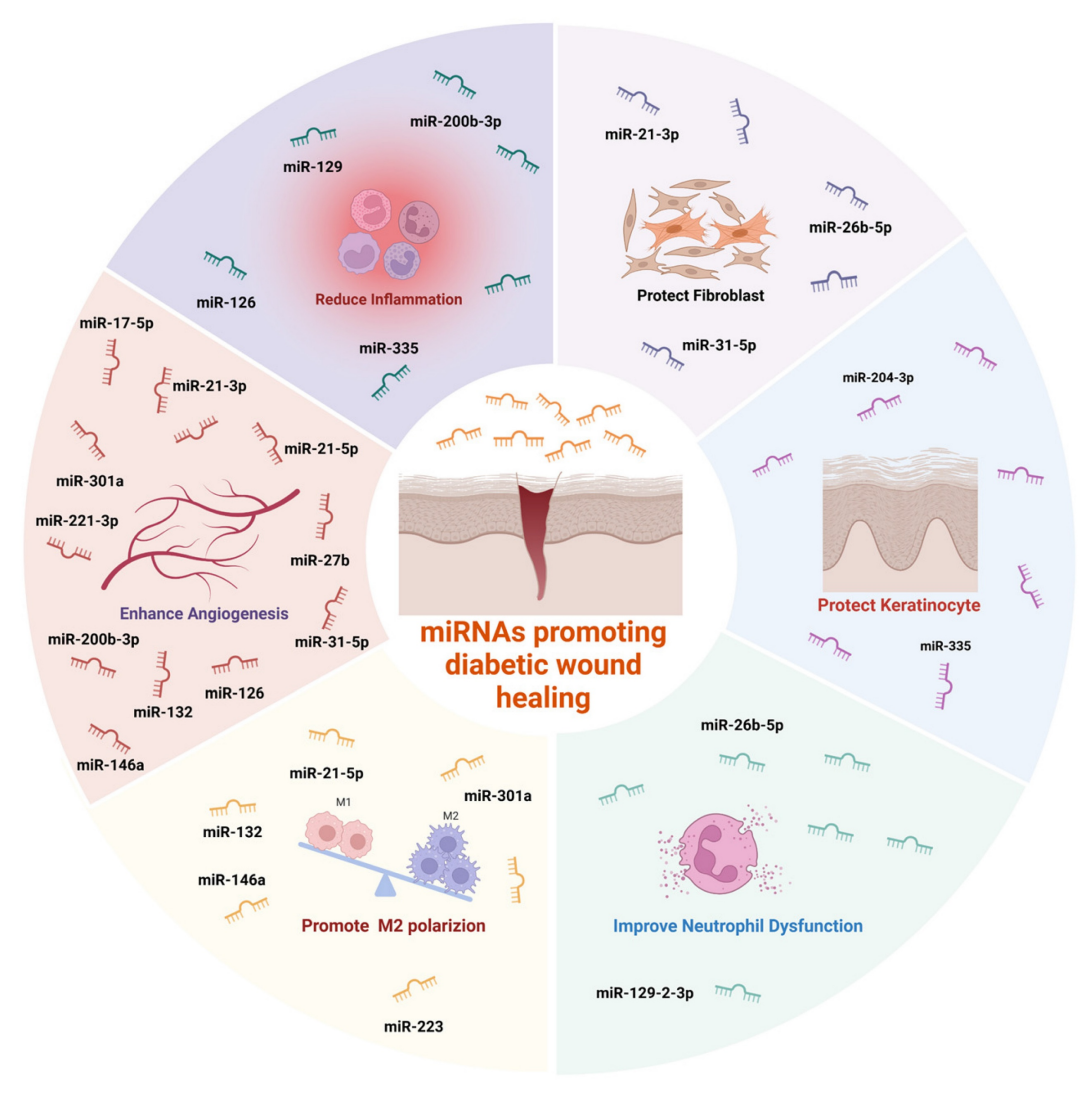
** miRNAs promoting diabetic wound healing.** Created with https://BioRender.com.

The functions of EVs are predominantly governed by their cargo, particularly microRNAs (miRNAs), which modulate gene expression by binding to target mRNAs 168. A growing body of evidence indicates that various miRNAs play promotive roles in diabetic wound repair (**Figure 5**) 169-177. The potential of EVs in this context continues to garner significant interest 176,177.

#### 4.2.6.1. miRNA in Stem Cell-Derived Extracellular Vesicles

ADSC-EVs are especially rich in diverse miRNAs compared to BMSC-EVs, endowing them with broader regulatory capabilities 119,178. These miRNAs promote cell proliferation, migration, angiogenesis, and immune modulation 119,179. For instance, miR-451a, highly expressed in ADSC-EVs, suppresses macrophage migration inhibitory factor (MIF), facilitating M2 polarization 179. Additionally, circRNAs and lncRNAs within ADSC-EVs modulate miRNA activity. mmu_circ_0001542, for example, upregulates miR-124-3p to enhance M2 polarization 180,181. Long non-coding RNA (lncRNA) H19, a competing endogenous RNA (ceRNA), modulates gene expression by sequestering miR-130b-3p, upregulating PPARγ and STAT3, and promoting an anti-inflammatory macrophage phenotype 182,183.

##### 4.2.6.2. Strategies for Regulating MiRNA in Extracellular Vesicles

Cell pre-conditioning offers a method to modify miRNA content in EVs. Hypoxia, for example, enhances mmu_circ_0001542 and miR-22-3p expression in ADSC-EVs, both of which regulate macrophage polarization 181,184,185. Selenium treatment has been shown to induce ADSCs to release EVs enriched in anti-inflammatory and pro-angiogenic miRNAs 186,187. Similarly, LPS stimulation upregulates miR-150-5p and let-7b, which promote M2 polarization 188,189. Additionally, Insulin-induced gene 1 (Insig1), a regulator of lipid metabolism, indirectly modulates the immunoregulatory properties of BMSC-EVs 190. Other stimuli, such as specific wavelength monochromatic light, may also influence EV-mediated immune functions, though this remains underexplored 191.

##### 4.2.6.3. Direct miRNA Delivery

Beyond cell-derived EVs, miRNAs can be directly incorporated into hydrogels for therapeutic use. Wu *et al.* embedded miR-301a into hydrogels, enhancing M2 polarization via the mTOR pathway 173. Similarly, Small interfering RNA (siRNA)—such as those targeting MMP9—have been used to adjust the M1/M2 macrophage ratio and reduce inflammation 192. Meanwhile, most studies on the immune modulation characteristics of post-intervention EVs are still in the basic research stage or directly applied to diabetic wounds, and research on EV-integrated hydrogels for diabetic wound applications remain relatively scarce.

### 4.3. Bioactive Substances

Although EVs have been widely applied in hydrogel-based delivery, their extraction and quality control are time-consuming and labor-intensive. Therefore, hydrogels loaded with bioactive substances that possess immune-regulatory capabilities have emerged as a focal point in diabetic wound research. Loading bioactive substances into hydrogels can prevent their rapid degradation *in vivo* while minimizing the waste or off-target effect caused by rapid release.

#### 4.3.1. Oxygen-Releasing Microspheres

**Figure 6:**
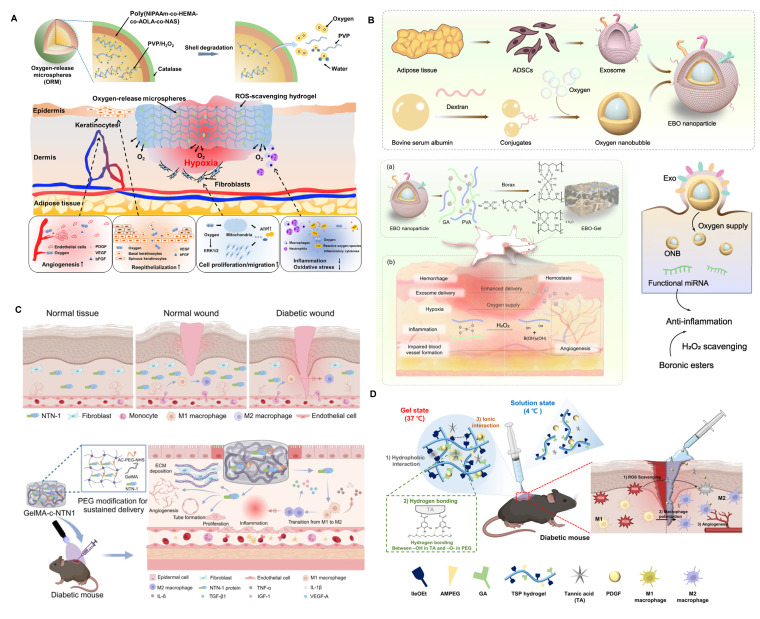
**Immunomodulatory hydrogel systems for delivering bioactive substances.** (A) The mechanisms of oxygen-release microspheres (ORMs) and accelerated wound healing by ORMs encapsulated in hydrogel. Adapted with permission from 195. Copyright 2021, American Association for the Advancement of Science. (B) The preparation and mechanisms of ADSC-derived exosome coated BSA-based oxygen nanobubbles. Adapted with permission from 196. Copyright 2024, Springer Nature. (C) The mechanisms of netrin-1 co-crosslinked hydrogel accelerated diabetic wound healing. Adapted with permission from 213. Copyright 2024, Elsevier. (D) The mechanisms of a thermo-responsive hydrogel and the wound healing process. Adapted with permission from 215. Copyright 2025, Royal Society of Chemistry.

Hypoxia is also a significant contributor to immune dysregulation within diabetic wounds. However, oxygen therapy has not yielded satisfactory results in diabetic wound healing, and even the explosive oxygen supply to the wound may exacerbate oxidative stress in the wound microenvironment. Therefore, providing a persistent and appropriate amount of oxygen to the wound is key to solving this issue 193,194. Guan *et al.* developed hydrogen peroxide-based oxygen-releasing microspheres and loaded them into ROS-scavenging hydrogels (**Figure 6A**) 195. This approach was shown to suppress M1 macrophage polarization and dampen pro-inflammatory signaling, thereby expediting wound resolution in diabetic models. However, even when loaded in ROS-scavenging hydrogels, the hydrogen peroxide-based oxygen release mechanism carries a certain risk of increasing oxidative stress in the diabetic microenvironment. Additionally, the uniform distribution of oxygen in wound tissue remains challenging, reducing therapeutic efficacy.

To overcome the limitations of H₂O₂-based oxygen release systems—which risk oxidative stress and uneven oxygenation—emerging strategies such as oxygen nanobubble-embedded hydrogels and hemoglobin-based oxygen carriers offer significant advantages. For instance, Han *et al.* developed an EV-coated oxygen nanobubble-laden hydrogel that effectively alleviated hypoxia while enhancing EV delivery, angiogenesis, and immunomodulation in a wound-healing model (**Figure 6B**) 196. Similarly, oxygen nanobubble-embedded Carbopol hydrogels have demonstrated stable and prolonged oxygen delivery—up to three weeks—improving wound healing without provoking oxidative injury 197.

Several studies have demonstrated the therapeutic potential of hemoglobin-based hydrogels in diabetic wound healing. For example, integrating hemoglobin with black phosphorus quantum dots into hydrogel microneedles enables near-infrared-triggered, on-demand oxygen release, leveraging the photothermal properties of BP to meet the dynamic oxygen demands of the diabetic wound microenvironment 198. Other approaches have modified red blood cells into Mn-based mineralized carriers, endowing them with sustained oxygen delivery and immunomodulatory capacity tailored for diabetic wounds 199. Nevertheless, hemoglobin-based systems still face important limitations. Hemoglobin is prone to oxidation and denaturation, which diminishes its oxygen-carrying capacity. Immunogenicity also remains a concern, particularly when hemoglobin is derived from xenogeneic sources. Moreover, environmental factors such as pH and temperature strongly affect hemoglobin stability, creating significant challenges for storage and long-term use 200.

#### 4.3.2. ROS-Scavenging Nanozymes

Nanozymes—synthetic enzyme mimetics—have drawn substantial interest owing to their distinctive enzyme-like functionalities. They not only efficiently scavenge ROS to alleviate oxidative stress but also catalyze the release of oxygen, improving hypoxic conditions and the immune microenvironment. This dual-regulatory function makes nanozymes highly promising tools for diabetic wound therapy. Li *et al.* developed a CaO_2_-based sustainable oxygen-releasing hydrogel, which regulates both angiogenesis and inflammation in diabetic wounds 201. MnO_2_ nanosheets, which are widely known for their ability to effectively scavenge ROS and generate oxygen, were incorporated into hydrogels by Tu *et al.* 202. This approach not only scavenged ROS in the diabetic wound environment, improving the hypoxic state, but also alleviated the dysregulation between neutrophils and macrophages. However, MnO_2_ and CaO_2_ exhibit pH-responsive rapid degradation, limiting the sustained effect of ROS clearance and oxygen release. Moreover, traditional nanozymes, while capable of scavenging existing ROS in diabetic wounds, cannot inhibit the ongoing generation of ROS. Therefore, the development of new nanozymes capable of continuously catalyzing ROS into oxygen has become a research hotspot. Li *et al.* and Zhao *et al.* utilized the peroxide-based MnCoO nanozyme properties to develop hydrogels that achieve this goal 203,204. These hydrogels not only continuously capture ROS in diabetic wounds but also generate oxygen via hydrogen peroxide while significantly inducing macrophage M2 polarization.

#### 4.3.3. Cytokines

Among the various delivery strategies mentioned above, whether regulating macrophage polarization or using other approaches, the focus is often on the changes modulating the balance between pro- and anti-inflammatory cytokine secretion. The network structure of hydrogels can stabilize cytokines, slow their release rate, and promote their prolonged action. Therefore, using hydrogels for the delivery of cytokines to reshape the immune landscape of diabetic wounds and enhance tissue regeneration is also a promising strategy.

Interleukins are a group of cytokines with highly dynamic and complex functions. They often interact through positive or negative regulation, playing pivotal roles in inflammation resolution and tissue repair. For example, IL-1, IL-6, and IL-17 act as potent inflammatory drivers, while IL-4 and IL-10 serve as hallmark anti-inflammatory mediators 205-208. In most cases, hydrogels are used to deliver anti-inflammatory cytokines like IL-4 and IL-33 to improve chronic inflammation in diabetic wounds 207,209. However, Yoon *et al.* loaded pro-inflammatory cytokines like IL-8 and macrophage inflammatory protein-3α into hydrogels to improve the delayed infiltration of inflammatory cells and promote wound healing 206.

Therefore, both pro-inflammatory and anti-inflammatory cytokines can promote healing in diabetic wounds, highlighting the importance of spatial-temporal-specific delivery of immune-regulatory agents during the physiological healing process of the wound. To achieve this, Tolouei *et al.* engineered a magnetically responsive delivery platform capable of orchestrating the early-stage recruitment of M1 macrophages via localized pro-inflammatory cytokine release, followed by the delayed administration of IL-4 and IL-10 under magnetic induction to steer macrophage polarization toward the M2 phenotype 205. However, simply time-specific immune regulation does not fully satisfy the need, therefore, researchers have combined IL-10 with VEGF and Ag nanoparticles cluster for layered packaging in hydrogels to achieve sequential release. This approach not only regulates immune modulation but also addresses infection and angiogenesis 208,210. Similar to using cationic polyethyleneimine-functionalized mesoporous polydopamine to remove excessive NETs from the wound, hydrogels can also be used to clear inflammatory chemokines from the wound (**Figure 6C**) 52,211. Recently, Emiroglu *et al.* developed a particulate hydrogel that can isolate IL-6 while simultaneously releasing VEGF, representing a novel strategy. In the future, hydrogels with spatiotemporal delivery and isolation capabilities hold great potential to further promote the healing of diabetic wounds 212.

#### 4.3.4. Peptides

In addition, bioactive peptides can also modulate the immune microenvironment. Netrin-1 has immune-regulatory properties, not only promoting inflammation in atherosclerosis but also alleviating inflammation following liver ischemia and reperfusion injury. Therefore, Shu *et al.* attempted to load Netrin-1 into GelMA hydrogels to modulate macrophage heterogeneity, improving the chronic inflammatory stage associated with low expression of Netrin-1 in diabetic wounds (**Figure 6C**) 213. The mitochondrial-targeted peptide SS31 can alleviate mitochondrial oxidative stress, promoting M2 macrophage polarization. To protect this bioactive peptide from rapid hydrolysis and ensure sustained release, Deng *et al.* loaded SS31 onto mesoporous polydopamine nanoparticles, which were further incorporated into hydrogels, achieving sustained immune reprogramming within the diabetic wound 214.

#### 4.3.5. Growth Factor

Growth factor-loaded hydrogels remain a cornerstone of bioactive wound dressings, aiming to restore impaired angiogenesis and immune balance in diabetic wounds. Among them, PDGF is the most clinically advanced; hydrogel-based delivery can overcome its short half-life and achieve sustained, localized release. For example, a hydrogel co-delivering PDGF and tannic acid promotes macrophage polarization toward M2 and enhanced angiogenesis in diabetic mouse wounds (**Figure 6D**) 215. Similarly, hierarchical hydrogels co-loaded with PDGF-BB and DNase I demonstrated dual benefits of promoting endothelial migration and degrading excessive neutrophil extracellular traps, thereby achieving dual-axis intervention in chronic inflammation and vascular insufficiency 216.

Fibroblast growth factors (FGF) have also shown promise. FGF-21-loaded heparin-poloxamer hydrogels significantly improved wound closure and angiogenesis compared with free FGF-21 in diabetic mice 217. In addition, recombinant human acidic FGF hydrogels mitigated NLRP3 inflammasome activation and promoted regenerative remodeling in diabetic ulcers 218.

Collectively, these studies demonstrate that growth factor-loaded hydrogels can both stimulate pro-regenerative pathways (e.g., VEGF/FGF signaling) and modulate immune responses, underscoring their dual role in correcting the ischemic and inflammatory barriers characteristic of diabetic wound healing.

## 5. Diabetes-Specific Efficacy of Immunomodulatory Hydrogel Strategies

Emerging evidence indicates that not all hydrogel-based immunomodulatory therapies exert uniform effects across wound types; rather, certain strategies confer diabetes-specific advantages by addressing pathologies unique to the diabetic milieu (**Table 3**). For example, MSC-derived EVs mitigate hyperglycemia-induced inflammation through miRNA-mediated suppression of NF-κB and NLRP3, while PDGF-BB hydrogels compensate for impaired PDGF signaling observed in diabetic wounds. Similarly, oxygen nanobubble and hemoglobin-based hydrogels overcome chronic hypoxia without aggravating oxidative stress, and DNase I-containing formulations directly target excessive NET formation—a pathological hallmark of diabetic ulcers. Other agents such as FGF21 offer additional metabolic benefits by improving insulin sensitivity and vascular function. These distinct features underscore that hydrogel therapies for diabetic wounds should not be evaluated solely by generic regenerative outcomes, but also by their capacity to address diabetes-specific immune, metabolic, and microenvironmental deficits.

## 6. Challenges and Future Perspectives

Diabetic wound healing is profoundly modulated by immune dynamics. Immune system imbalance, such as macrophage polarization dysfunction, excessive neutrophil activation, T cell dysfunction, and oxidative stress dysregulation, significantly impedes wound repair under conditions of persistent chronic inflammation. This review systematically summarizes the key immunological abnormalities within diabetic wounds and discusses the immunoregulatory potential of hydrogels loaded with various biological components. It focuses on how hydrogels can enhance the regenerative niche through cell-based delivery strategies, extracellular vesicles, and bioactive factors. Despite the remarkable potential of immune-regulatory hydrogels in diabetic wound-related research, their clinical translation still faces several challenges:

### 6.1. Translational Barriers in Hydrogel

Scalability of hydrogel fabrication remains one of the most critical obstacles for clinical translation. Although hydrogels have shown considerable efficacy as carriers for immunomodulatory agents in preclinical models, few formulations have been produced under conditions compatible with clinical-scale production. Laboratory preparation typically relies on small-batch, manually prepared hydrogels, which are poorly aligned with industrial requirements for reproducibility, cost-effectiveness, and regulatory compliance. Advanced fabrication approaches, such as microfluidics or 3D printing, further complicate scalability by introducing challenges of process reproducibility and material standardization 225.

An essential yet under-discussed aspect of advancing hydrogel-based immunomodulatory platforms toward clinical application is the necessity for comprehensive safety evaluations. First, while numerous hydrogels show promise in preclinical studies, translating these materials safely to humans mandates rigorous assessment of biocompatibility, immunotoxicity, and off-target effects. For instance, a recent review on clinical hydrogel applications identified 139 clinical trials involving hydrogel dressings or implants, yet many failed to report on long-term immune responses or systemic safety outcomes 226. Second, beyond acute toxicity, hydrogels must undergo extended observation for potential chronic inflammation, immunogenic reactions, or unintended immune activation, especially when delivering cells, biologics, or immune-active cargo. Materials designed for immune modulation may induce unexpected feedback responses if not carefully characterized in relevant large-animal or human-like models.

Regulatory approval constitutes another major bottleneck. Clinical translation of hydrogels must comply with international standards (e.g., ISO 10993), ensuring biocompatibility, sterility, and biosafety. In addition, hydrogel systems delivering immunologically active substances require rigorous evaluation under investigational new drug applications (INDAs) and subsequent regulatory pathways, such as new drug applications (NDAs) or biologics license applications (BLAs) 227. This multilayered regulatory framework, involving multiple agencies, substantially prolongs the time required for bench-to-bedside translation.

Physicochemical composition further impacts manufacturability. Polymer selection, crosslinking chemistry, and water content not only determine therapeutic properties but also affect sterilization compatibility and batch-to-batch reproducibility. For instance, conventional sterilization processes can partially degrade hydrogel networks, while high water content complicates storage and shelf life 228,229.

Encouragingly, technological solutions are beginning to emerge. For example, Del Río *et al.* demonstrated that 3D printing can be leveraged to scale up biohybrid hydrogel structures for T cell culture, enhancing reproducibility, cell infiltration, and nutrient transport. Such approaches may provide a blueprint for future immunomodulatory hydrogel platforms applicable to diabetic wound healing 230.

In addition to scalability, safety, and regulatory compliance, hydrogel systems for diabetic wound healing must also meet disease-specific design requirements. The diabetic wound microenvironment is characterized by persistent hyperglycemia, high levels of AGEs, excessive ROS, and protease overactivity, all of which can compromise the stability and function of hydrogel dressings. For example, glycation can alter polymer crosslinking and mechanical integrity, while elevated ROS and MMPs may accelerate premature degradation, leading to uncontrolled release of therapeutic payloads. To address these challenges, new generations of hydrogels are being developed with anti-glycation modifications, ROS- and MMP-responsive linkers, and multimodal protective chemistries that allow spatiotemporal control of degradation.

Collectively, these challenges underscore that the successful translation of hydrogels will require not only advances in scalable and standardized manufacturing, but also the integration of regulatory compliance and material innovations to ensure clinical feasibility.

### 6.2. Barriers to Delivering Cells

Hydrogel-based delivery systems for cells, particularly stem cells, hold considerable therapeutic promise but face substantial translational hurdles. First, cell viability and retention in the harsh diabetic wound microenvironment (characterized by oxidative stress, high protease activity, and hypoxia) remain problematic. Hydrogels must be engineered not only to protect embedded cells but also to support their paracrine or immunomodulatory functionality over time. Second, regulatory and clinical translation face intensified scrutiny when viable cells are delivered: formulations must ensure cell identity, biosafety, absence of tumorigenicity, and functional consistency under GLP/GMP conditions. This complexity increases both development timelines and regulatory burden.

Taken together, although cell delivery via hydrogels enhances therapeutic potential, clinical translation demands robust solutions for scalable production, functional stability, and regulatory compliance.

### 6.3. Challenges in Clinical-Grade Preparation and Translation of EVs

Despite extensive research on EVs, no EV-based therapeutics have yet received regulatory approval for clinical use, largely due to challenges in production scale, product consistency, safety, and cost. Several major barriers remain to be addressed before EVs can be translated into clinical-grade products.

Achieving high-yield, bioactive EV production remains a persistent difficulty. Conventional cells secrete limited amounts of EVs, and while external stimulation can increase output, it often alters the molecular composition and biological activity of EVs. For instance, silicate stimulation of endothelial progenitor cells enhances EV secretion but simultaneously modifies angiogenic factor content 231. At present, there is no consensus on standardized interventions for boosting EV yield without compromising functionality 231-233.

At the same time, laboratory ultracentrifugation methods are inadequate for Good Manufacturing Practice (GMP) standards. Efforts to scale up have focused on combining tangential flow filtration, ultracentrifugation with density gradients, and size exclusion chromatography to generate high-quality EVs 234. However, reproducibility and cost remain limiting factors for industrial application.

The cellular origin of EVs poses another challenge. For mesenchymal stem cell-derived EVs, batch-to-batch variability and serum-derived impurities hinder reproducibility, while EVs derived from tumor cell lines raise concerns regarding oncogenicity and immune modulation. Standardization of EV potency and quality control—including particle number, size distribution, total protein, lipid, RNA content, and marker profiling—remains an unmet requirement for regulatory approval 235,236.

EVs are vulnerable to damage even at -80 °C, as ice crystal formation can impair biological activity. Although cryoprotectants such as phosphate-buffered saline with sucrose or trehalose have been explored, stability varies across EV sources, highlighting the absence of unified strategies for long-term preservation 232. Following administration, EVs suffer from rapid clearance and suboptimal tissue targeting. Engineering approaches, including surface modification with targeting peptides, represent promising strategies to improve biodistribution and functional delivery. While most preclinical studies confirm EV safety in animal models, comprehensive data on biodistribution, pharmacokinetics, and immunological effects in humans are still lacking, necessitating further clinical investigation.

Together, these barriers underscore the urgent need for standardized production protocols, improved storage and delivery strategies, and rigorous safety assessments to accelerate the clinical translation of EV-based therapeutics. When combined with hydrogel platforms, these challenges in EV standardization, storage, and delivery become even more complex, underscoring the need for integrated solutions that address both material and biological barriers in clinical translation.

### 6.4. Limitations of Cytokines, Peptides, Growth Factors, and Antibodies

Diabetic wounds are characterized by elevated levels of inflammatory cytokines such as IL-1β, IL-6, and TNF-α, which collectively hinder the healing process. Anti-inflammatory cytokine therapies can act through mechanisms including direct neutralization, receptor blockade, receptor modulation, or selective depletion of target cells. Among these, cytokine-targeting antibodies neutralize inflammatory mediators without binding to cell-surface receptors, offering longer-lasting effects with potentially fewer off-target consequences 237. However, compared with cytokines, peptides, and growth factors, antibodies are large proteins with limited tissue penetration and diffusion capacity. In contrast, small-molecule inhibitors can achieve similar immunomodulatory effects while exhibiting superior tissue permeability. For example, blockade of the IL-1 pathway using IL-1 receptor antagonists has been shown to markedly restore the immune microenvironment and promote healing in diabetic and chronic wound models 223.

Many current hydrogel formulations suffer from limited spatiotemporal precision in therapeutic release, often resulting in transient or poorly timed immune modulation. In addition, these bioactive molecules are prone to rapid degradation and typically have short half-lives *in vivo*, resulting in transient or unstable immunomodulatory effects. Their delivery also carries the risk of systemic diffusion beyond the wound site, which may trigger off-target effects or systemic immunosuppression. This represents a significant safety concern that remains insufficiently investigated in current preclinical models.

### 6.5. Limited Precision in Delivery

Many current hydrogel formulations suffer from limited spatiotemporal precision in therapeutic release, often resulting in transient or poorly timed immune modulation. Although smart hydrogels responsive to pH, temperature, enzymes, or ROS offer enhanced precision, their performance can be inconsistent due to patient heterogeneity and dynamic changes in wound conditions. Developing patient-specific, adaptive hydrogel systems remains a critical priority.

### 6.6. Immunogenicity Risks of Xenogeneic Hydrogel Materials

The immunogenicity of xenogeneic hydrogel materials represents a critical yet frequently overlooked barrier to clinical translation. Natural polymers such as alginate, collagen, and chitosan are widely used in hydrogel systems due to their biocompatibility and gel-forming properties. However, their biological origin inherently carries the risk of undesirable immune activation. For instance, alginate formulations vary in purity and may contain endotoxins or protein contaminants that stimulate macrophages via TLR4 signaling, thereby initiating a pro-inflammatory cascade 238. Similarly, collagen and decellularized ECM hydrogels can retain residual antigens or DAMPs, triggering both cellular and humoral immune responses even after decellularization 239.

Chitosan illustrates the complexity of immunogenicity, as its effects vary depending on molecular weight, degree of deacetylation, and residual endotoxin levels. These parameters can determine whether chitosan elicits an inflammatory or reparative response 240. To mitigate such risks, advanced purification, chemical modification, and endotoxin removal technologies are increasingly being applied to natural polymer systems such as alginate and chitosan 241. In parallel, synthetic or semi-synthetic alternatives—including PEG-based and peptide-derived hydrogels—offer more predictable immunological profiles and align more closely with regulatory expectations 242.

Nevertheless, the hyperinflammatory environment of diabetic wounds amplifies the consequences of even low-level immune stimulation by xenogeneic matrices, potentially undermining the therapeutic efficacy of encapsulated immunomodulatory agents. Future research should therefore prioritize the development of standardized immunogenicity assays—such as *in vitro* human immune cell co-culture and *in vivo* diabetic wound models—to systematically evaluate both hydrogel carriers and their therapeutic cargo. Ensuring that the delivery vehicle itself does not exacerbate inflammation is essential for the successful clinical translation of hydrogel-based immunotherapies.

### 6.7. Complexity and Target Selection in Immune Modulation

Immune responses in wound healing shift from early activation to later resolution, yet static immunomodulatory approaches cannot accommodate this transition. Although staged-release hydrogels have been proposed, standardized protocols for timing and dosing remain undeveloped. In diabetic wounds, immune dysregulation involves multiple cell types, but most studies disproportionately target macrophage polarization, while other pathways—such as neutrophil extracellular trap clearance or T cell regulation—are rarely explored. Moreover, few comparative studies evaluate different strategies to identify the most relevant immune targets. Future efforts should therefore integrate spatiotemporal delivery control with broader and more systematic target selection to optimize hydrogel-based immunotherapies for diabetic wound healing.

### 6.8. Patient Heterogeneity and Limitations of Preclinical Models

A major limitation of current research lies in the insufficient consideration of patient heterogeneity and the reliance on inadequate preclinical models. Most studies employ rodent models such as mice and rats, which differ markedly from humans in wound healing mechanisms, while porcine models offer closer approximation but remain logistically difficult to manipulate. In contrast, clinical patients with T1D and T2D display distinct metabolic and immune profiles that shape wound healing responses differently. Furthermore, diabetic foot ulcers can be stratified into neuropathic, ischemic, or mixed phenotypes, each driven by distinct pathological mechanisms. For instance, hydrogels engineered with neuroregenerative properties may be preferentially effective in neuropathic ulcers but show limited benefit in ischemic wounds, where angiogenic factor-loaded formulations such as VEGF- or angiopoietin-based hydrogels may be more suitable. Addressing this heterogeneity requires both the development of more predictive and manipulable platforms—such as organ-on-a-chip systems—and the incorporation of patient stratification into preclinical and clinical trial design. Ultimately, modular hydrogel systems tailored to wound-specific phenotypes will be essential for ensuring clinical efficacy of hydrogel-based immunotherapies.

### 6.9. Limited Exploration of Immune Cell Interactions

Most research has focused on macrophage polarization, while the roles of additional immune populations—including T lymphocytes, neutrophils, and so on- remain underexplored. Future studies should aim to understand how hydrogels influence the broader cell populations to achieve more precise immune modulation. Additionally, a novel immune modulation strategy involves the crosstalk between non-immune cells and immune cells that play pivotal roles in wound resolution to regulate immune responses in diabetic wounds. For instance, fibroblasts can release Macrophage Colony-Stimulating Factor (M-CSF) to enhance macrophage phagocytosis.

### 6.10. Future Research Directions

To better promote the application of immunomodulatory hydrogels in diabetic wounds, we proposed three feasible scientific research hypotheses: immune sensing, artificial intelligence drive, and modular hydrogel platform, which may affect the next generation of hydrogel-based diabetic wound immunotherapy.

#### 6.10.1. Real-Time Immunosensing Hydrogels

To move beyond general observations, future research must focus on testable hypotheses that integrate hydrogel design with dynamic immune regulation and patient-specific needs. One promising avenue is the development of hydrogels capable of real-time immune sensing, extending beyond current systems that mainly monitor pH or glucose. By incorporating biosensors for inflammatory cytokines (e.g., IL-1β, TNF-α), such platforms could provide direct insights into the immunological status of diabetic wounds and guide timely therapeutic interventions 243,244.

#### 6.10.2. Integration with AI-Driven Wearable Devices

Another direction involves coupling hydrogels with AI-driven wearable devices. These integrated systems would not only monitor biochemical and biophysical indicators but could also trigger hydrogels to release immunomodulatory cargos in response to detected abnormalities. Such “closed-loop” wound management would allow both predictive monitoring and on-demand therapy, thereby improving therapeutic precision 245,246.

#### 6.10.3. Modular and Patient-specific Hydrogel Design

Finally, advances in modular and customizable hydrogel design are expected to enable therapies tailored to specific wound phenotypes. Hydrogels could be engineered with interchangeable modules that address neuropathic versus ischemic ulcers, or T1D versus T2D immune profiles, thus aligning with patient stratification strategies. Combining modularity with intelligent sensing and AI integration could ultimately establish personalized hydrogel platforms that restore immune balance and accelerate diabetic wound healing.

## 7. Conclusion

Diabetic wounds present persistent clinical challenges due to immune dysregulation and chronic inflammation. Engineered immunomodulatory hydrogel delivery systems hold significant promise by enabling targeted and sustained release of therapeutics—including cells, EVs, and bioactive molecules—to restore immune balance. These platforms regulate macrophage polarization, suppress neutrophil overactivation, and promote angiogenesis, thereby accelerating wound healing. Among them, MSC-EVs and immune cell-based therapies have shown particular potential, offering low immunogenicity, high stability, and versatile immunoregulatory functions.

Despite encouraging progress, several obstacles remain. Clinical translation is hampered by scalability, regulatory hurdles, and safety concerns, while limitations of delivered cargos, the need for precise spatiotemporal control, and complex hydrogel-cargo interactions further complicate application. Moreover, although most strategies demonstrate broad regenerative efficacy, their diabetes-specific advantages are not fully understood. For example, MSC-EVs may exert unique immunomodulatory effects in diabetic wounds, but the mechanisms and wound subtype specificity require clarification. Future studies should focus on treatment strategies targeting such diabetes-specific mechanisms—such as immune microenvironment modulation, glycemic regulation, AGEs resistance, and stability under hyperinflammatory or high-MMP conditions—and integrating them into hydrogel design. Addressing these challenges will be critical for advancing hydrogel-based immunotherapies into effective clinical treatments for diabetic wound management.

## Figures and Tables

**Table 1:** Cells-loaded immunomodulatory hydrogels for diabetic wound healing applications.

Cell type	Materials	Treatment	Model	Results	Days for wound healing	Refs.
BMSCs	NIPAM, PAA, APS, TEMED	Thermosensitive hydrogel encapsulating BMSCs	Diabetic mice (C57BL/6) full-thickness cutaneous wound	Inhibiting M1 polarization; Promoting angiogenesis	Unhealed, 35 days	67
BMSCs	N-chitosan, HA-ALD, ADH	Self-healing hydrogel encapsulating BMSCs	Diabetic rat (SD) foot skin wound	Enhancing M1-to-M2 macrophage polarization; Promoting angiogenesis	15 days	68
BMSCs	Chitosan, PEG, Glutaraldehyde, Glycerol, Ethanol	Porous hydrogel encapsulating FGF21 pretreated BMSC	Diabetic rat (SD) full-thickness cutaneous wound	Suppressing apoptotic and inflammatory genes expression	16 days	69
ADSCs	HB-PEGDA, Thiolated gelatin	Injectable hydrogel encapsulating allogeneic ADSCs	Diabetic mice (db/db) full-thickness cutaneous wound	Suppressing infiltration of inflammatory cells; Promoting angiogenesis	15 days	70
ADSCs	GelMA	3D bioprinting hydrogel encapsulating curcumin and ADSCs	Diabetic athymic nude mice (nu/nu) full thickness cutaneous wound	Suppressing AGEs-related inflammatory;Promoting angiogenesis	21 days	71
hUCMSCs	GelMA, ZnCl_2_,Chitosan-catechol,DTT	Hybrid hydrogel encapsulating HUMSCs	Diabetic mice (db/db) full-thickness cutaneous wound	Downregulating inflammatory factors (TNF-α and IL-1β); Promoting angiogenesis and collagen deposition	14 days	72
hUCMSCs	Peptide RADA16-I; Peptide KLT; Peptide RGD	Self-assembled nanopeptide hydrogels encapsulating hUCMSCs spheroids	Diabetic mice (NOD/SCID) full-thickness cutaneous wound	Downregulating inflammatory factors; Promoting angiogenesis	10 days	73
WJMSCs	PF-127; SAP	Injectable and temperature-sensitive hydrogel encapsulating WJMSCs	Diabetic rat (SD) full-thickness cutaneous wound	Enhancing M1-to-M2 macrophage polarization; Promoting angiogenesis	Unhealed(residual wound area<10%), 14days	74
HUVEC^vegf165+^	γ-PGA-SH; γ-PGA-GMA; RGDC; LAP	3D printed all-peptide hydrogel encapsulating HUVEC^vegf165+^	Diabetic rat (SD) full-thickness cutaneous wound	Reducing wound inflammation; Improving angiogenesis, ECM remodeling, and cell adhesion	14 days	75
Macrophage	Alginate	Alginate hydrogels encapsulating macrophage	Diabetic mice (C57BL/6) full-thickness cutaneous wound	Any polarized macrophage subtype and their secretome similarly promotes diabetic wound healing in 10 days	10 days(M2c)	76
hiPSC-SMCs		3D collagen scaffolds encapsulating hiPSC-SMCs	Male athymic nude mice full-thickness cutaneous wound	Promoting angiogenesis; Increased number of total and M2 type macrophages.	10 days	77

**Table 2:** EVs-loaded immunomodulatory hydrogels for diabetic wound healing applications.

EVs source	Materials	Treatment	Model	Results	Days for wound healing	Refs
BMSCs-EVs	GelMA, DOPA, EDC, NHS	Adhesive *in situ* photo-crosslinked hydrogel encapsulating BMSCs-EVs	Diabetic rat (SD) full-thickness cutaneous wound	Downregulating inflammatory factors (IL-6); Promoting angiogenesis	14 days	101
BMSCs-EVs	Chitosan, CMC, acrylic acid	Antibacterial and self-healing hydrogel encapsulating BMSCs-EVs	Diabetic rat (SD) full-thickness cutaneous wound	Enhancing M1-to-M2 macrophage polarization; Promoting angiogenesis	14 days	102
ADSCs-EVs	4-Arm-PEG-Thiol, AgNO_3_, MWCNTs	Injectable, adhesive, self-healing hydrogels encapsulating ADSCs-EVs	Diabetic mice (Balb/c) full-thickness cutaneous wound	Downregulating inflammatory factors (IL-6, TNF-α); Promoting angiogenesis	14 days	103
ADSCs-EVs	Porcine cardiac tissue	ECM hydrogel encapsulating ADSCs-EVs	Diabetic mice (ICR) full-thickness cutaneous wound	Downregulating inflammatory factors (IL-6, TNF-α);Promoting angiogenesis	14 days	104
ADSCs-EVs	HB-PEG, HA-SH	*In situ* formed hydrogel encapsulating hypoxic pretreated ADSCs-EVs	Diabetic rat (SD) full-thickness cutaneous wound	Downregulating TNF-α; Preventing scar formation	Unhealed(residual wound area<10%), 21 days	105
hUCMSCs-EVs	Alginate, HDAC7-derived 7-amino-acid peptide, CaCl_2_	Alginate hydrogel encapsulating hUCMSCs-EVs and 7A	Diabetic rat full-thickness cutaneous wound	Downregulating inflammatory factors (IL-6, IL-1β, TNF-α);Promoting angiogenesis	Unhealed(residual wound area<10%), 18 days	106
FSMSCs-EVs	PVP, SiW	Self-healing, adhesive, and antibacterial hydrogel encapsulating FSMSCs-EVs	Diabetic mouse full-thickness excisional wound	Enhancing M1-to-M2 macrophage polarization;Promoting angiogenesis	12 days	107
M2-EVs	HAMA, PVA, LAP, PDA, Dil	Photosensitive hydrogel microneedles encapsulating M2-EVs and PDA nanoparticles	Diabetic rat (SD) full-thickness cutaneous wound	Enhancing M1-to-M2 macrophage polarization;Promoting angiogenesis	14 days	108
M0-EVs	CMC, HA, NBT, DMF	Hydrogel with M0-EVs	Diabetic rat (SD) full-thickness cutaneous wound	Enhancing M1-to-M2 macrophage polarization; Releasing VEGF	14 days	109
Treg-EVs	PF-127	Thermoresponsive hydrogel encapsulating Treg-EVs	Diabetic mice (C57BL/6) full-thickness cutaneous wound	Enhancing M1-to-M2 macrophage polarization;Reducing IL-6 production;Enhancing skin and vascular endothelial cell migration	Unhealed(residual wound area>60%), 7 days	110
AT-EVs	Egg whites	Hydrogel with both radial topological and biological cues encapsulating AT-EVs	Diabetic mice (C57BL/6) full-thickness cutaneous wound	Enhancing M1-to-M2 macrophage polarization;Promoting angiogenesis	14 days	111
PRP-EVs	PF-127	Thermoresponsive hydrogel encapsulating PRP-EVs	Diabetic mice (db/db) full-thickness cutaneous wound	Inhibiting fibroblast ferroptosis; Increasing M2 macrophage	Unhealed(residual wound area≈20%), 14 days	112
PLT-EVs	Gelatin, alginate, Reduced graphene oxide	Photothermally responsive hydrogel encapsulating PLT-EVs	Diabetic rat (Wistar) full-thickness cutaneous wound	Enhancing M1-to-M2 macrophage polarization	14 days	113
PLT-EVs	ADM, MA	Photo-crosslinking and fast-gelling hydrogel encapsulating PLT-EVs	Diabetic rat (SD) full-thickness cutaneous wound	Enhancing M1-to-M2 macrophage polarization;Promoting angiogenesis	21 days	114
G-EVs	HA, MES, EDC, HOBT, ADH, OSA, Mg^2+^	Injectable hydrogel encapsulating G-EVs^DM^	Diabetic mice full-thickness cutaneous wound	Enhancing M1-to-M2 macrophage polarization;Promoting angiogenesis	14 days	115
Apoptotic EVs	GelMA, LAP	Photo-crosslinking hydrogel encapsulating apoptotic EVs	Diabetic mice (db/db) full-thickness cutaneous wound	Enhancing M1-to-M2 macrophage polarization;Enhancing the endothelial cell and fibroblast function	14 days	116

**Table 3:** Diabetes-specific efficacy of hydrogel-based delivery of immunomodulatory strategies for diabetic wound healing.

Therapy / Cargo	Diabetes-Specific Features	Key Mechanisms	Refs.
BMSCs/ADSCs/hUCMSCs	Dual action on inflammation and angiogenesis tailored to hyperglycemic milieu	M1→M2 polarization, endothelial protection	219
BMSC-EVs	Suppress NF-κB/NLRP3 activation; promote M1→M2 polarization even under hyperglycemia	miR-146a-5p, miR-223 regulate inflammatory pathways	220,221
hUCMSC-EVs	Enhance angiogenesis and immune regulation in diabetic models	miR-17-5p promotes angiogenesis and anti-inflammation	221,222
O₂ Nanobubbles/Hb Hydrogels	Continuous oxygen release; lower oxidative stress risk in hyper-ROS environment	ROS/MMP-responsive O₂ release	197,198
EV-O₂ Nanobubbles	Relieve hypoxia and improve EV uptake under diabetic wound conditions	Sustained oxygen supply + enhanced EV stability	196
ROS-Scavenging Nanozymes	Specifically mitigate oxidative stress in diabetic wounds	Promote M2 polarization, anti-inflammatory effects	201-204
IL-1Ra (matrix-bound)	Provides long-acting local immunoregulation; suppresses persistent IL-1 activity	IL-1 pathway blockade	223
FGF21	Corrects metabolic imbalance and vascular inflammation unique to diabetes	Corrects metabolic imbalance and vascular inflammation unique to diabetes	217,218
PDGF-BB	Counteracts suppressed PDGF signaling in diabetes	Counteracts suppressed PDGF signaling in diabetes	216,224
DNase I	Degrades excess NETs, targeting diabetes-specific high NETosis	Degrades excess NETs, targeting diabetes-specific high NETosis	216
